# Regulation of the interferon-gamma (IFN-γ) pathway by p63 and Δ133p53 isoform in different breast cancer subtypes

**DOI:** 10.18632/oncotarget.25635

**Published:** 2018-06-26

**Authors:** Sunali Y. Mehta, Brianna C. Morten, Jisha Antony, Luke Henderson, Annette Lasham, Hamish Campbell, Heather Cunliffe, Julia A. Horsfield, Roger R. Reddel, Kelly A. Avery-Kiejda, Cristin G. Print, Antony W. Braithwaite

**Affiliations:** ^1^ Pathology Department, University of Otago, Dunedin, New Zealand; ^2^ Maurice Wilkins Centre for Molecular Biodiscovery, University of Auckland, Auckland, New Zealand; ^3^ Department of Molecular Medicine and Pathology, University of Auckland, Auckland, New Zealand; ^4^ Priority Research Centre for Cancer Research, Innovation and Translation, School of Biomedical Sciences and Pharmacy, Faculty of Health and Medicine, The University of Newcastle, Calvary Mater Hospital, Waratah NSW, Australia; ^5^ Children’s Medical Research Institute, The University of Sydney, Westmead, NSW, Australia

**Keywords:** TP53, TP63, isoforms, immune response, breast cancer

## Abstract

The *TP53* family consists of three sets of transcription factor genes, *TP53*, *TP63* and *TP73*, each of which expresses multiple RNA variants and protein isoforms. Of these, *TP53* is mutated in 25-30% of breast cancers. How *TP53* mutations affect the interaction of *TP53* family members and their isoforms in breast cancer is unknown. To investigate this, 3 independent breast cancer cohorts were stratified into 4 groups based on oestrogen receptor (ER) and *TP53* mutation status. Using bioinformatic methodologies, principal signalling pathways associated with the expression of *TP53* family members were identified. Results show an enrichment of IFN-γ signalling associated with *TP63* RNA in wild type *TP53* (wt*TP53*), ER negative (ER-) tumours and with *Δ133TP53* RNA in mutant *TP53* (m*TP53*) ER positive (ER+) tumours. Moreover, tumours with low IFN-γ signalling were associated with significantly poorer patient outcome. The predicted changes in expression of a subset of RNAs involved in IFN-γ signalling were confirmed *in vitro*. Our data show that different members of the *TP53* family can drive transcription of genes involved in IFN-γ signalling in different breast cancer subgroups.

## INTRODUCTION

The *TP53* family consists of three sets of transcription factors *TP53*, *TP63* and *TP73*, which are involved in cell development, homeostasis and response to stress (reviewed in [1]). *TP53* plays a central role in maintaining the integrity of the genome, therefore it is not surprising that *TP53* is frequently mutated in most human cancers [2, 3]. Unlike *TP53*, mutations are uncommon in *TP63*, but it is overexpressed in a subset of basal cell and squamous cell carcinomas of the head and neck, due to chromosomal amplification, and elevated levels of *TP63* RNA have been reported in a number of different tumour types (reviewed in [4]). Similarly, mutations are uncommon in *TP73* but it is overexpressed in high grade breast cancers [5]. All three genes use alternative promoters and RNA splicing resulting in expression of multiple RNA variants and protein isoforms [1, 6]. This results in a complex array of isoforms encoding proteins containing an intact N-terminal transactivation domain that are capable of activating p53 target genes (TAp53, TAp63 and TAp73) [1, 6] and N-terminally truncated proteins including ΔNp53, ΔNp63, ΔNp73 that may serve as antagonists of their TA counterparts [1, 6]. To add to the complexity, all three family members encode C-terminal variants [1, 6].

Clinically, breast cancers are categorised into three groups including oestrogen receptor positive (ER+), *HER2* amplified, and Triple Negatives (TNBC, lacking expression of ER, progesterone receptor (PR) and HER2) [7]. Around 25-30% of breast cancers harbour a somatic mutation in the *TP53* gene [8]. Prevalence of *TP53* mutations is subtype dependent and are more frequent in TNBCs compared to other subtypes [7, 9]. In general, breast cancers with *TP53* mutations are known to have a poor clinical outcome [7, 9]. In addition to mutations, expression of *TP53* isoforms and other family members have been associated with poor patient outcome. Examples include: (1) elevated levels of *Δ40TP53* RNA in TNBCs [10]; (2) elevated levels of *Δ133TP53* RNA in other breast cancers [11]; and (3) elevated *ΔNTP63* RNA levels in high risk breast cancers [12]. Additionally it has been shown that Δ133p53 promotes invasion of breast cancer cell lines [11] and ΔNp63 promotes a cancer stem-like cell in TNBCs [12]. Conversely TAp63 suppresses metastasis in breast cancer [13] and loss of TAp73 or upregulation of ΔNp73 has been associated with increased angiogenesis in breast cancer [14]. This complex transcriptional pattern and potential interplay among *TP53* family members makes translating *TP53* mutation status into clinical utility difficult.

In this paper we investigated associations between *TP53* mutations, *TP53* isoform expression and gene expression of other family members in 3 breast cancer cohorts using bioinformatics. Analyses of the data show that interferon-gamma (IFN-γ) signalling is associated with *TP63* RNA expression in ER negative (ER-) tumours without *TP53* mutations and with expression of *Δ133TP53* isoform in ER+ tumours with *TP53* mutations. These results suggest that TAp63 regulates IFN-γ signalling in ER- wt*TP53* tumours and that Δ133p53 regulates this pathway in ER+ m*TP53* tumours. This prediction was confirmed by *in vitro* experiments. Thus, TAp63 and Δ133p53 can carry out similar functions in different breast cancer subtypes depending on *TP53* and ER status. Our analyses also demonstrate that low expression of interferon-gamma (IFN-γ) signalling is associated with poor patient outcome independent of subtype.

## RESULTS

### *TP53* RNA expression associates with the regulation of cell proliferation and immune response processes only in ER- wt*TP53* breast cancers

Three breast cancer cohorts were analysed for *TP53* mutation frequency, type and distribution. *TP53* was mutated in 24% (n=421) of the invasive breast cancer cohort from The Cancer Genome Atlas (TCGA), 23% (n=251) from GSE3494 and 36% (n=61) from GSE61725 (Supplementary Figure 1A). Missense mutations were most common across the 3 cohorts (50 – 64%), followed by frameshift mutations (14 – 27%) and nonsense mutations (12 – 32%) respectively. The cohorts were further divided into ER+ and ER- groups. Consistent with previous reports [7, 9], *TP53* mutations were significantly more frequent in ER- tumours (P < 0.05, chi-square test), (Supplementary Figure 1A-1B). The majority of the missense mutations were clustered in the DNA binding domain (DBD, codons 95-289, Supplementary Figure 1C) whereas the frameshift and nonsense mutations tended to be scattered throughout the coding sequence [15] (Supplementary Figure 1C). These 3 cohorts were selected for further analyses as they are representative of frequency, type and distribution of *TP53* mutations generally found in breast cancer.

**Figure 1:**
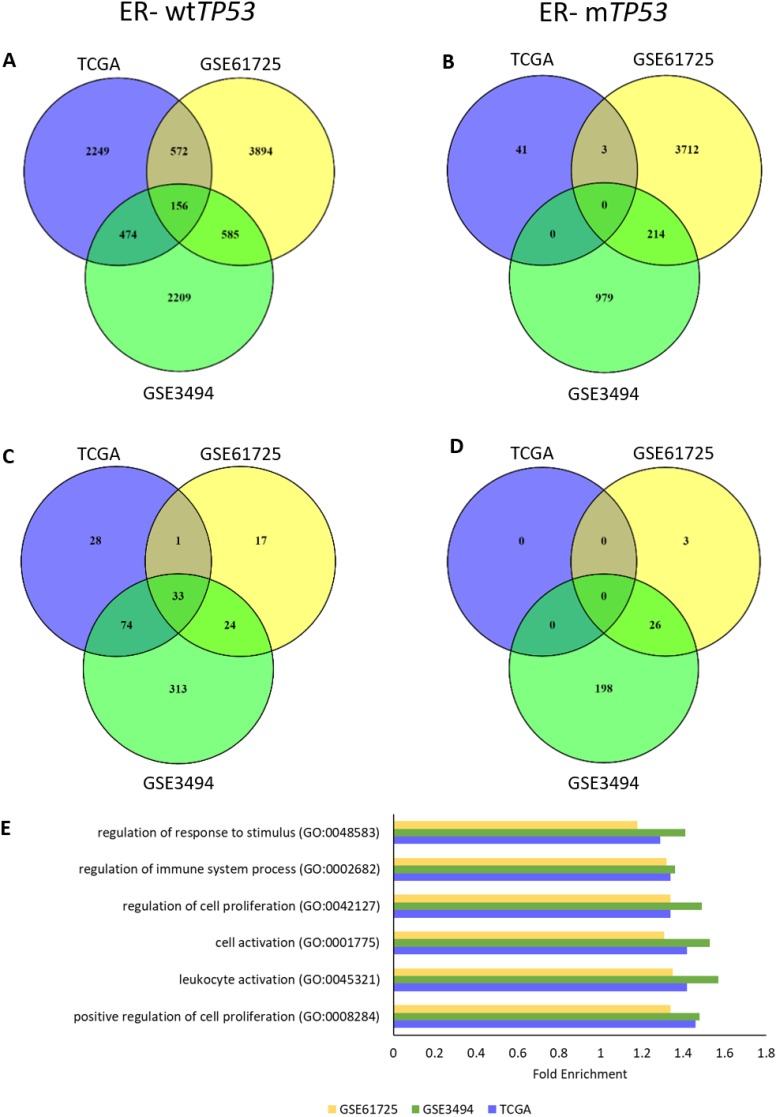
*TP53* RNA expression associates with cell proliferation and immune response in ER- wt*TP53* tumours **(A-B)** Number of overlapping RNAs associated (ρ = ± 0.4) with *TP53* RNA expression in the 3 cohorts (TCGA, GSE61725 and GSE3494). (A) ER- wt*TP53* (TCGA – n = 24, GSE61275 – n = 11 and GSE3494 – n = 15). (B) ER- m*TP53* (TCGA – n = 51, GSE61275 – n = 14 and GSE3494 – n = 19). **(C-D)** Number of overlapping over-represented GO biological processes associated with *TP53* RNA expression (Bonferroni corrected P<0.05) in the 3 cohorts (TCGA, GSE61725 and GSE3494). (C) ER- wt*TP53*. (D) ER- m*TP53*. **(E)** List of Top 5 enriched common GO biological processes associated with *TP53* RNA expression in ER- wt*TP53* tumours between the three cohorts.

To determine if expression of specific gene sets varied with *TP53* gene expression, each cohort was divided into 4 subgroups, based on ER status and *TP53* mutation: ER+ wt*TP53*, ER+ m*TP53*, ER- wt*TP53* and ER- m*TP53*. RNAs associated with *TP53* RNA expression in each of the subgroups across the three cohorts were determined using Spearman’s correlation (ρ = ± 0.4). In ER- wt*TP53* tumours, there was an overlap of 156 RNAs associated with wt*TP53* (Figure 1A). However, there were essentially no common RNAs associated with *TP53* in any of the other subgroups (Figure 1B, Supplementary Figure 2A and 2B). Lack of common RNAs associated with *TP53* RNA expression in these subgroups was not due to lack of variation or low RNA expression of *TP53* (Supplementary Figures 3 and 4).

To identify gene ontology (GO) in the RNAs associated with *TP53* RNA expression, a statistical overrepresentation test using PANTHER [16] was performed. Results showed that there were 33 common GO biological processes associated with *TP53* RNA expression in ER- wt*TP53* tumours (Figure 1C). Of these, the top 5 are involved in the regulation of cell proliferation and immune system processes (Figure 1E and Supplementary Table 2). On the other hand, there were no common GO biological processes associated with *TP53* RNA expression in the other subgroups (Figure 1D and Supplementary Figure 2C and 2D).

### *TP53* transcript level variation cannot account for loss of geneset association in mutant *TP53* breast tumours

**Figure 2:**
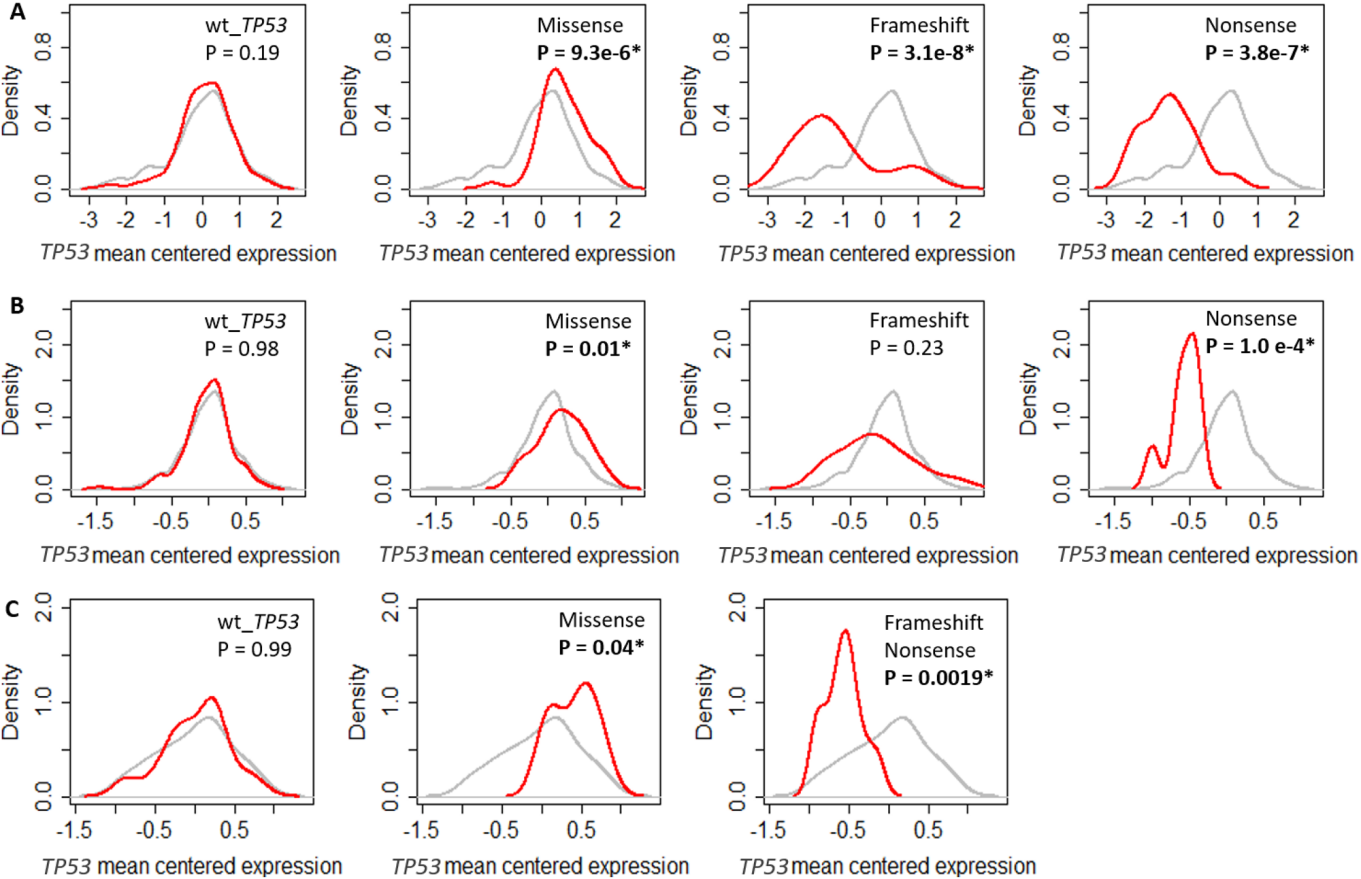
Impact of *TP53* mutation on *TP53* RNA expression in different breast cancer cohorts Density plots showing the levels of mean centered *TP53* RNA levels in **(A)** TCGA. **(B)** GSE3494. **(C)** GSE61725. The grey line shows the density of the mean centered *TP53* RNA levels of all the tumours in individual cohorts. The red line shows the density of the mean centered *TP53* RNA levels in breast tumours with either wild type *TP53* or missense, frameshift and nonsense mutations, in individual cohorts. Significant shift in the distributions of *TP53* RNA levels if P < 0.05 determined using two-sided Kolmogorov-Smirnov test.

Mutations in *TP53* can affect gene transcription and transcript stability, which could explain the lack of common GO biological processes associated with *TP53* RNA expression in m*TP53* tumours. To test this, the tumours were divided into 4 groups based on *TP53* mutation status. The amount of *TP53* RNA in each of the 4 groups was quantitated. *TP53* RNA expression in the GSE61725 cohort was combined for tumours with FS and nonsense mutations due to limited numbers (FS tumours = 3). Results showed that tumours with missense mutations had increased levels of *TP53* RNA (P < 0.05, two-sided Kolmogorov-Smirnov test) (Figures 2A – 2C) whereas tumours with nonsense mutations had noticeably decreased levels of *TP53* RNA (Figure 2). Tumours with FS mutations also had substantially decreased levels of *TP53* RNA in the TCGA and GSE61725 cohorts and a similar trend was observed in the GSE3494 cohort (Figure 2). Reduction in *TP53* RNA expression in the presence of nonsense or FS mutations in *TP53* may be explained by nonsense mediated decay [17]. Thus, loss of expression of m*TP53* RNA cannot account for the loss of association of the GO biological processes except in cases of tumours with FS or nonsense mutations.

### Overlap in GO biological processes associated with wt*TP53* RNA expression in ER- wt*TP53* and ER- tumours with frameshift and nonsense mutations

To explain the loss of association of the GO biological processes with *TP53* RNA expression in the presence of missense *TP53* mutations, 2 other possibilities were considered: (1) the GO biological processes are directly associated with *TP53* RNA expression (i.e. driven by wtp53), which is lost when *TP53* is mutated or (2) they are indirectly associated with *TP53* (i.e. driven by something else) but blocked by a ‘gain-of-function’ (GOF) activity of mp53. To test which of these hypotheses better explains the data, we took advantage of the loss of *TP53* RNA levels in tumours with FS and nonsense (fsn) mutations from the large TCGA cohort. For the purpose of the analysis we consider these fsn mutations to be “functionally” null. Spearman’s correlation (ρ = ± 0.4) followed by a statistical overrepresentation test [16] was performed and GO biological processes were compared from tumours with wt*TP53*, m*TP53* and fsn*TP53*. Results showed that there was an overlap of 37 GO biological processes between ER- wt*TP53* and ER- fsn*TP53* tumours, again involved mainly in regulation of cell proliferation and immune system processes (Supplementary Figure 5 and Supplementary Table 3), but there were no common GO processes in the ER- m*TP53* subgroups (Supplementary Figure 5). These results suggest that the GO biological processes involved in regulation of cell proliferation and immune response are indirectly associated with *TP53* RNA expression, but inhibited by GOF missense p53 mutants.

### *TP63* RNA expression is associated with immune response processes in ER- wt*TP53* tumours

**Figure 3:**
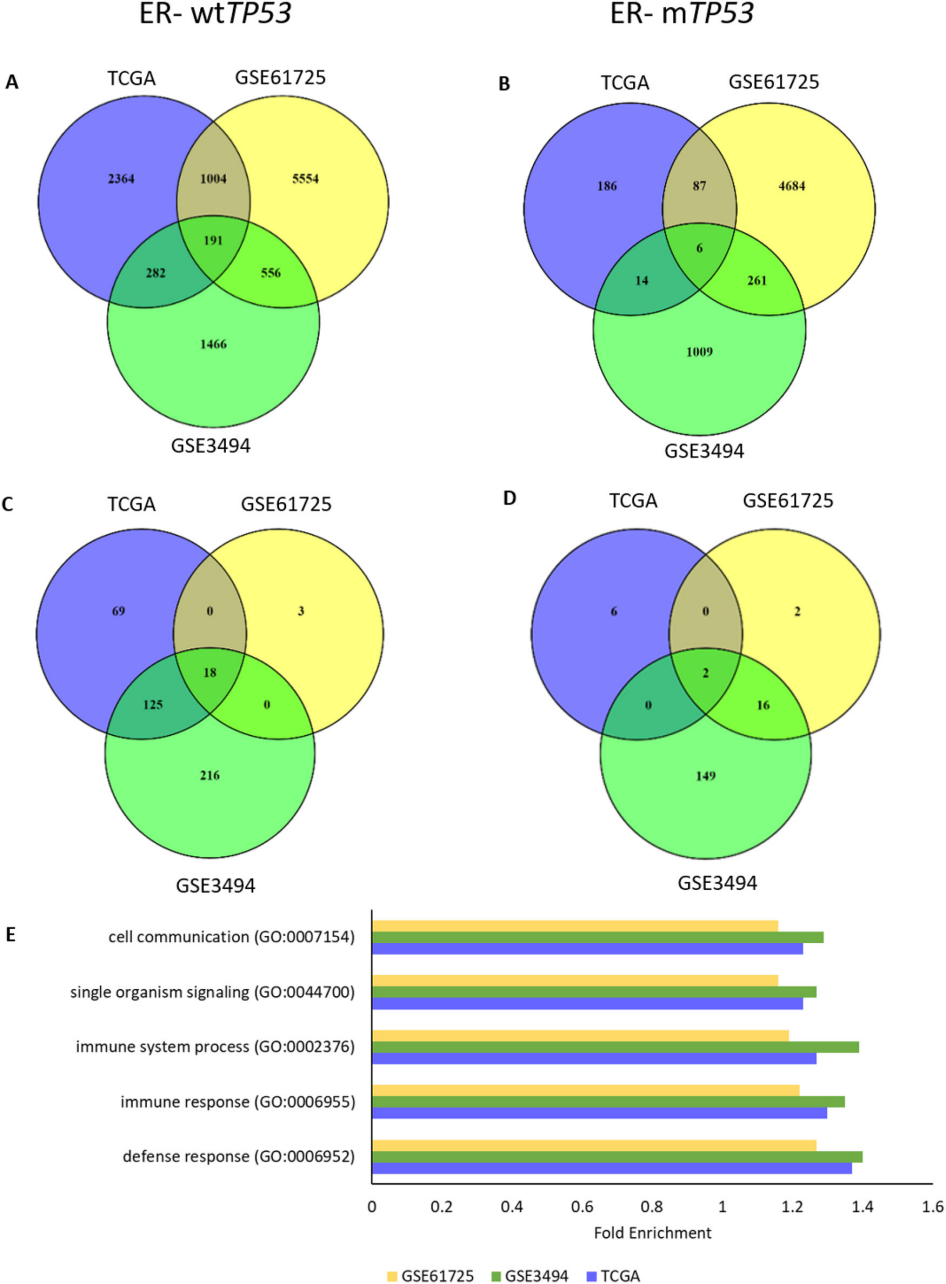
*TP63* RNA expression associates with regulation of immune response processes in ER- wt*TP53* tumours **(A-B)** Number of overlapping RNAs associated (ρ = ± 0.4) with *TP63* RNA expression between the 3 cohorts (TCGA, GSE61725 and GSE3494). (A) ER- wt*TP53* (TCGA – n = 24, GSE61275 – n = 11 and GSE3494 – n = 15). (B) ER- m*TP53* (TCGA – n = 51, GSE61275 – n = 14 and GSE3494 – n = 19). **(C-D)** Number of overlapping over-represented GO biological processes associated with *TP63* RNA expression (Bonferroni corrected P<0.05) between the 3 cohorts (TCGA, GSE61725 and GSE3494). (C) ER- wt*TP53*. (D) ER- m*TP53*. **(E)** List of Top 5 enriched GO biological processes associated with *TP63* RNA expression in ER- wt*TP53* tumours.

**Figure 4:**
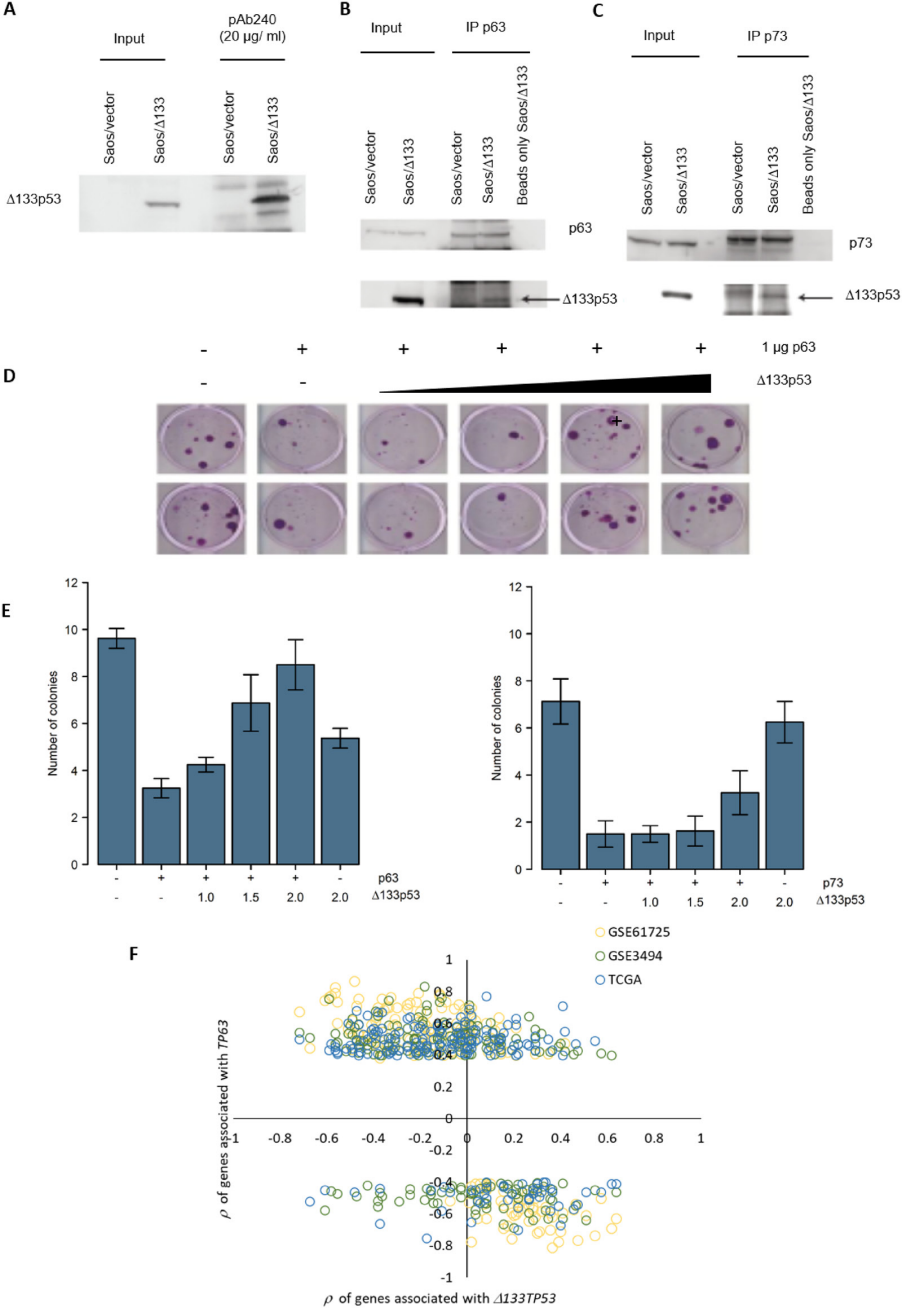
Δ133p53 inhibits TAp63 and TAp73 and *Δ133TP53* RNA expression is inversely correlated with *TP63* associated genes in ER- wt*TP53* tumours **(A)** Δ133p53 was immunoprecipitated with the p53 conformation specific antibody pAb240 and immunoblotted with the same antibody. **(B-C)** TAp63 and TAp73 were immunoprecipated and immunoblotted with pAB240. **(D)** An example of the colony formation assay carried out in H1299 cells, co-transfected with a TAp63 expression construct and increasing amounts of a Δ133p53 expressing plasmid, along with the CMVNeo plasmid. **(E)** Quantification of G418 resistant colonies after transfection with TAp63 and Δ133p53 (left graph) and TAp73 and Δ133p53 (right graph). The bars represent the mean and error bars are ± S.D. of 8 biological replicates. **(F)** Association of the 191 genes associated with *TP63* RNA expression in ER- wt*TP53* tumours from TCGA, GSE61725 and GSE3494 and with *Δ133TP53* RNA expression in ER- wt*TP53* tumours from GSE61725.

One GOF mechanism that has been described is the ability of missense p53 mutants to physically interact with, and inactivate, TAp63 and TAp73 [18–22]. Thus we investigated whether the genes involved in immune response in the ER- wt*TP53* tumours could be driven by TAp63 or TAp73. RNAs associated with *TP63* and *TP73* RNA expression in the 4 subgroups were determined using Spearman’s correlation (ρ = ± 0.4). In the ER- wt*TP53* tumours, there were 191 common genes (Figure 3A) and 18 over-represented GO biological processes (Figure 3B, Supplementary Table 4) associated with *TP63* RNA expression, but this association was not observed in the other subgroups (Figures 3B and 3D, Supplementary Figure 6). The top 5 over-represented GO biological processes are associated with immune system processes (Figure 3E, Supplementary Table 4) including the interferon-gamma (IFN-γ) mediated signalling pathway and cell communication (Supplementary Table 5). In the ER- m*TP53* tumours there were only 6 common RNAs associated with *TP63* RNA (Figure 4B) and these were not representative of immune gene sets (Figure 3D). Lack of common genes across the 3 subsets was not due to lack of variation or low expression of *TP63* RNA (Supplementary Figure 7). There were no common GO biological processes associated with *TP73* RNA expression in all the 4 subgroups (Supplementary Figures 8 and 9) which is likely due to low levels of *TP73* RNA in the GSE3494 cohort (Supplementary Figure 10). Taken together, these results suggest that in ER- wt*TP53* tumours, *TP63* is associated with IFN-γ signalling, which is lost in tumours with m*TP53*.

### *Δ133TP53* RNA is inversely correlated with the RNA expression of *TP63* and its associated genes in ER- wt*TP53* tumours

As well as mutant p53, several p53 isoforms are associated with cancer progression, particularly Δ133p53 which promotes proliferation, invasion, angiogenesis, and induces inflammatory gene transcription, including genes involved in *IFN-γ* signalling [23–25]. Thus, it seems possible that Δ133p53 might behave like missense p53 mutants. In support of this, Δ133p53 was immunoprecipitated with the conformation specific antibody pAb240, indicating that Δ133p53 has a mutant-like conformation (Figure 4A) and in co-immunoprecipitation experiments Δ133p53 was found in complex with TAp63 and TAp73 (Figure 4B - 4C). To test whether Δ133p53 inhibits TAp63 and TAp73 functions, colony forming assays were done. *TP53* null H1299 cells were transfected with TAp63 or TAp73 along with a plasmid conferring resistance to the G418 antibiotic and increasing amounts of a Δ133p53 expression plasmid. Cells were incubated in the presence of G418 and the number of surviving colonies counted. Results showed that TAp63 and TAp73 repressed colony formation by approximately 3 fold compared to the control (Figure 4D-4E), which is rescued by co-expression of Δ133p53. These results suggest that, like missense mutant p53, Δ133p53 can inhibit TAp63 and TAp73 functions. Thus, in some contexts, *Δ133TP53* may inhibit TAp63 activity and therefore its expression might be negatively correlated with TAp63 regulated transcripts (e.g. those in the IFN-γ signalling pathway).

To investigate this, we combined *Δ133TP53* RNA expression determined by RT-qPCR with gene expression from microarray data for the tumours in GSE61725. This analysis was limited to the tumours in the GSE61725 cohort, since RNA-seq is unable to detect expression of individual *TP53* RNA variants [26] (TCGA cohort) and the Affymetrix U133 A and B microarrays do not have probe-sets in the region unique to *Δ133TP53* (GSE3494) [26]. Consistent with the hypothesis, results showed that genes positively correlated with *TP63* RNA expression in ER- wt*TP53* tumours in all 3 cohorts, were negatively correlated with *Δ133TP*53 RNA expression in GSE61725 (Figure 4F).

### *Δ133TP53* RNA regulates genes involved in cytokine mediated signalling in ER+ m*TP53* tumours

We next asked whether expression of *Δ133TP53* RNA was associated with immune response genes in any of the other subgroups by carrying out correlation analyses in the GSE61725 cohort. There were 80 GO biological processes associated with *Δ133TP53* RNA expression (Supplementary Figure 11A) irrespective of *TP53* mutation status. However, there were 18 unique GO biological processes associated with *Δ133TP53* RNA expression in ER+ m*TP53* tumours (Supplementary Figure 11A). These were involved in the regulation of cell adhesion, cell communication and regulation of immune system processes (Supplementary Table 6). Moreover, there were 69 RNAs common among the three cohorts that were associated with *TP63* RNA expression in ER- wt*TP53* tumours and *Δ133TP53* RNA expression in ER+ m*TP53* tumours from GSE61725 (Supplementary Figure 11B). These 69 RNAs were involved in IFN-γ mediated and cytokine mediated signalling (Supplementary Table 7). These results suggest that in the presence of ER signalling and mutant *TP53*, *Δ133TP53* is involved in positively regulating cytokine mediated signalling, especially the IFN-γ signalling pathway.

### Regulation of IFN-γ signalling genes by *TP63* or *Δ133TP53 in vitro*

Our bioinformatic results suggest that *TP63* drives expression of cytokine mediated genes in ER- wt*TP53* tumours and *Δ133TP53* drives these in ER+ m*TP53* tumours. To test this *in vitro*, we selected 8 genes known to be involved in IFN-γ signalling and which were associated with either the expression of *TP63* or *Δ133TP53* in the respective subgroups (Supplementary Figure 11B). These include *STAT6, GBP2, IRF2, IL6ST, CXCR6, IL13RA1, JAK2* and *PIK3R3.*

To confirm that oestrogen signalling is important for altering the expression of the selected genes, two cell lines were used, MCF7 that are ER+ wt*TP53* and T47D that are ER+ m*TP53*. The cell lines were transfected with an siRNA to *ESR1*, RNA collected and RT-qPCR performed. Expression of 4/8 and 5/8 genes (p < 0.05, student’s t-test) significantly increased in MCF7 and T47D cells relative to the control siRNA (Supplementary Figure 12A and 12B). Next, cells were starved of estradiol for 72h, followed by addition of 100 nM β-estradiol, RNA collected 24h later, and RT-qPCR performed. Expression of 7/8 and 5/8 genes were significantly altered (p < 0.05, student’s t-test) in both cell lines (Supplementary Figure 12A and 12B). Furthermore, *ESR1* knock down and β-estradiol addition largely showed inverse patterns (Supplementary Figure 12A and 12B). In addition, oestrogen signalling also significantly altered expression of *TP63* and *Δ133TP53* (Supplementary Figure 12A and 12B).

**Figure 5:**
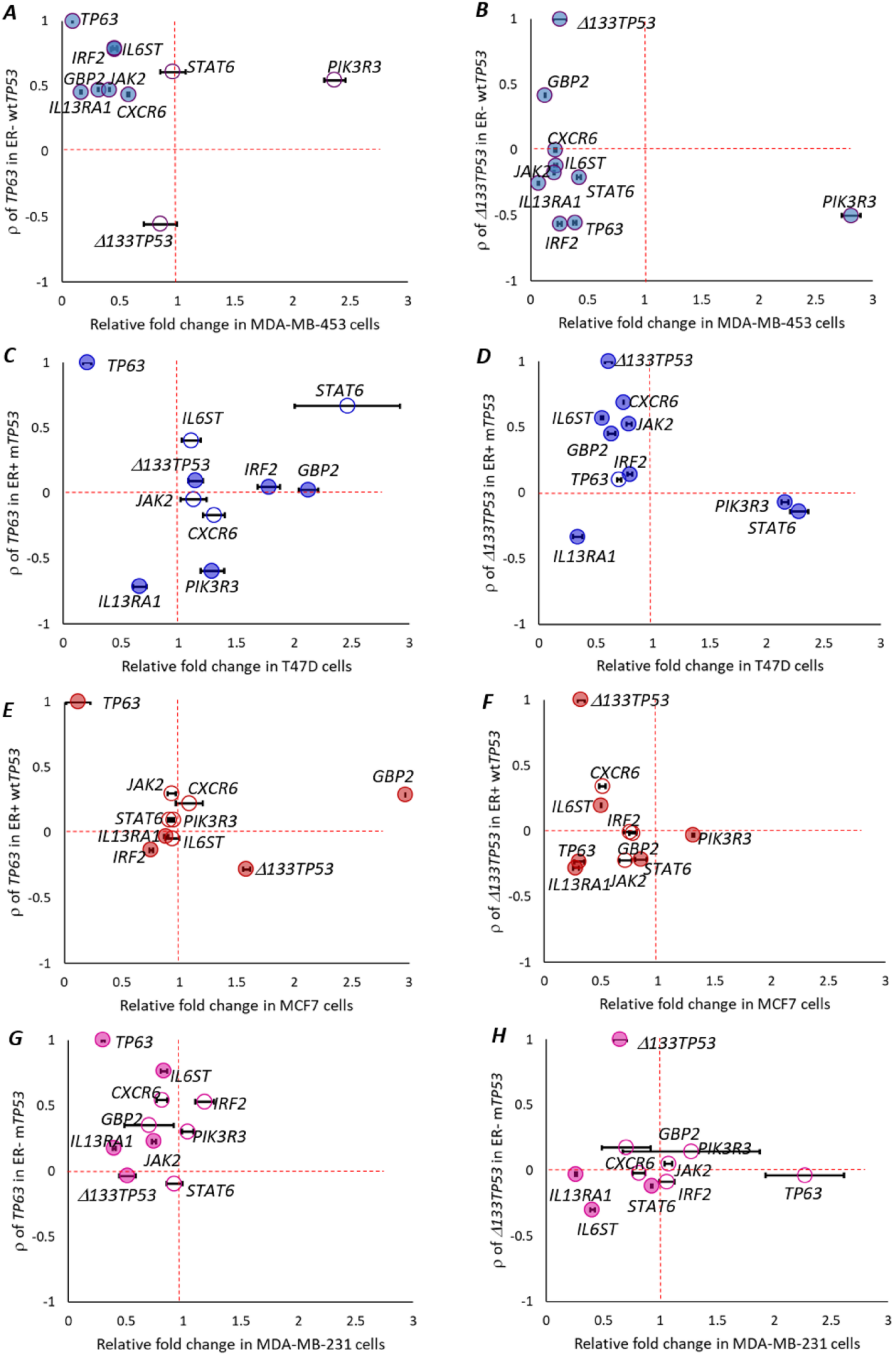
*TP63* or *Δ133TP53* regulates IFN-γ signalling *in vitro* Relative fold change in the expression of candidate IFN-γ signaling pathway genes, *TP63* and *Δ133TP53*, 48h post treatment with siRNA targeting either *TP63* or *Δ133TP53* in **(A-B)** MDA-MB-453 cells that are ER- wt*TP53*. **(C-D)** T47D cells that are ER+ m*TP53*. **(E-F)** MCF7 cells that are ER+ wt*TP53*. **(G-H)** MDA-MB-231 cells that are ER- m*TP53*. A-H. (A) Relative fold change after si-*TP63* treatment in MDA-MB-453 cells. (B) Relative fold change after si-*Δ133TP53* treatment in MDA-MB-453 cells. (C) Relative fold change after si-*TP63* treatment in T47D cells. (D) Relative fold change after si-*Δ133TP53* treatment in T47D cells. (E) Relative fold change after si-*TP63* treatment in MCF7 cells. (F) Relative fold change after si-*Δ133TP53* treatment in MCF7 cells. (G) Relative fold change after si-*TP63* treatment in MDA-MB-231 cells. (H) Relative fold change after si-*Δ133TP53* treatment in MDA-MB-231 cells. (A-H) Association of RNA expression of candidate IFN-γ signaling pathway genes on the y-axis and relative fold change 72h post transfection on the x-axis. Genes with positive correlation are above the horizontal dotted line and those that are negatively correlated are below the horizontal dotted line. Genes that are upregulated relative to the si-Ctrl are to the right of the vertical dotted line and downregulated genes are to the left of the vertical dotted line. Genes that are changed significantly are represented with filled colored circles, ● = significant, ○ = not significant (student’s t-test, p < 0.05). Levels were measured in three biological replicates for each treatment.

To test if the levels of the 8 selected genes changed by altering the levels of *Δ133TP53* or *TP63*, we transfected MDA-MB-453 (ER- wt*TP53*), MCF7, T47D and MDA-MB-231 (ER- m*TP53*) cells, with siRNAs targeted to *TP63* or *Δ133TP53*, collected RNA and performed RT-qPCR. In the MDA-MB-453 cells, expression of genes that were positively correlated with *TP63* expression in ER- wt*TP53* tumours (*IL6ST*, *IRF2*, *GBP2*, *JAK2*, *IL13RA1*, *CXCR6* – above the horizontal dotted line in Figure 5A) were reduced post *TP63* knockdown (left side of the vertical dotted line in Figure 5A, p <0.05, student’s t-test), *STAT6* did not change, and *PIK3R3* did not follow the predicted trend. *Δ133TP53* expression did not change. Thus, 6/8 genes were altered by *TP63* knockdown in the MDA-MB-453 cells. Although not predicted from the tumour analyses, knockdown of *Δ133TP53* also resulted in reduced expression of 7/8 genes as was *TP63* (Figure 5B).

In confirmation of these results, MCF7 cells were starved of estradiol for 72h, to create an ER- wt*TP53* environment. These cells were treated 24h post estradiol starvation with siRNAs targeted to *TP63* or *Δ133TP53* either individually or in combination with siRNA targeted to *ESR1*. RNA was collected 48h post transfection, and RT-qPCR performed. In the estradiol starved MCF7 cells, in line with our bioinformatic predictions, 6/8 genes were significantly altered upon *TP63* knockdown (Supplementary Figure 13A). As predicted, knockdown of *Δ133TP53* in the estradiol starved MCF7 cells had much less effect (Supplementary Figure 13B). Similarly, in estradiol starved MCF7 cells, when *ESR1* and *TP63* were knocked down in combination, the predicted bioinformatic trend was followed, where 6/8 genes were significantly altered (Supplementary Figure 13C). However, knockdown of *ESR1* and *Δ133TP53* altered expression of all genes, unlike what was observed in the tumour analyses (Supplementary Figure 13D).

In the T47D cells, expression of genes that were positively correlated with *Δ133TP53* expression in ER+ m*TP53* tumours, 5/8 genes (*IL6ST*, *CXCR6*, *GBP2, IRF2* and *JAK2*) were reduced post *Δ133TP53* knockdown (Figure 5D), whilst *STAT6* and *PIK3R3* that were negatively correlated with *Δ133TP53* expression in ER+ m*TP53* were increased after Δ1*33TP53* knockdown (Figure 5D). *IL13RA1* did not follow the predicted trend and *TP63* did not change. By contrast, knockdown of *TP63* had no effect on expression of these genes (Figure 5C). Also in line with our bioinformatic predictions, expression of the 8 selected genes was not significantly altered after knockdown of either *TP63* or *Δ133TP53* in the MCF7 cells (Figure 5E-5F) or in MDA-MB-231 cells (Figure 5G-5H).

Thus in conclusion, the *in vitro* results mostly support the bioinformatic predictions providing evidence that in ER- wt*TP53* tumours, *TP63* regulates expression of IFN-γ signalling genes and *Δ133TP53* does so in ER+ m*TP53* tumours.

### Reduced IFN-γ-mediated signalling is associated with poor survival outcome

**Figure 6:**
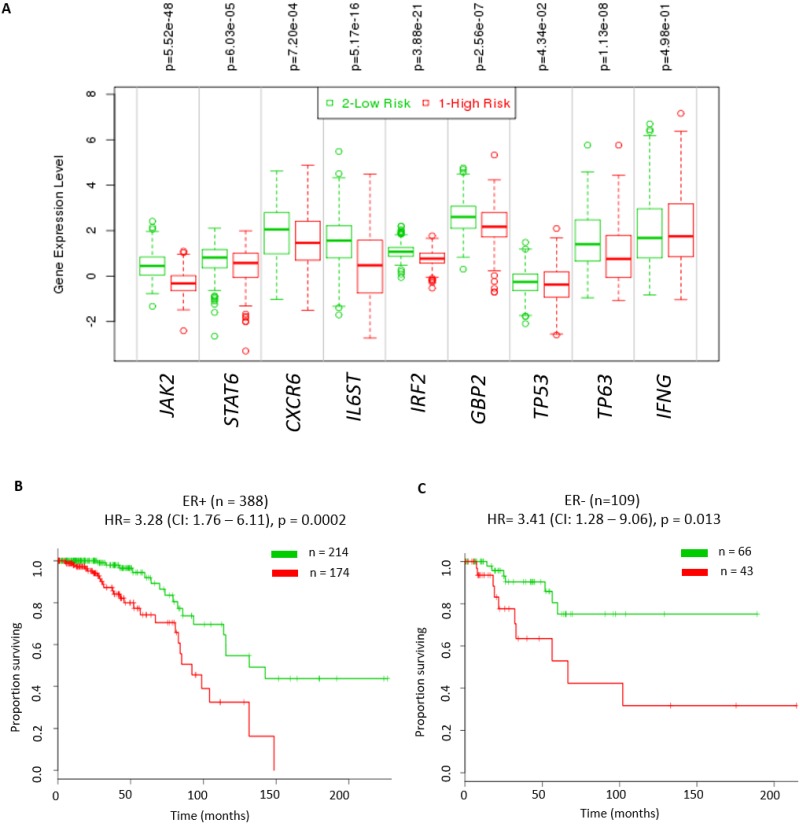
Significant prognostic value of IFN-γ mediated signalling in breast cancer patients Kaplan – Meier plot of high risk versus low risk tumours stratified by expression of IFN-γ, *TP63*, *TP53* and in combination with the 6 candidate genes (*JAK2*, *STAT6*, *CXCR6*, *IL6ST*, *IRF2*, and *GBP2*) representative of IFN-γ signalling from the TCGA cohort. **(A)** Log_2_RNA expression levels of these 9 genes in high risk and low risk breast tumours from the TCGA cohort using t-test (^*^ - p<0.05 was considered significant). High risk groups were those patients that had reduced disease free survival compared to low risk group patients. **(B)** ER positive tumours (hazard ratio (HR) = 3.28 (CI: 1.76 – 6.11), p = 0.0002). **(C)** ER negative tumours (hazard ratio (HR) = 3.41 (CI: 1.28 – 9.06), p = 0.013). All analyses were done using SurvExpress web resource [28].

Effective IFN-γ signalling is critical for activation, clonal expansion, memory development and for efficient natural killer (NK)-cell-mediated cytotoxicity [27]. Thus, it is possible that patients with tumours that have reduced IFN-γ signalling are likely to be associated with poor clinical outcome. In order to test the prognostic value of IFN-γ mediated signalling, we performed survival analyses using RNA expression data for *IFNG, TP63*, *TP53* independently and in combination with the 6 validated genes (*JAK2*, *STAT6*, *CXCR6*, *IL6ST*, *IRF2*, and *GBP2*). These analyses were performed using the Breast Invasive Carcinoma TCGA cohort in the SurvExpress database [28] since this dataset had the largest number of tumours (n = 502). Initially we assessed if expression of *IFNG* alone or in combination with expression of *TP53* and *TP63* was able to identify patients with poor outcome. We found that despite there being significantly lower log_2_ RNA expression of *IFNG* and *TP63* and higher log_2_ RNA expression of *TP53* (P ≤0.05) in the high risk group, these molecules did not have prognostic value in either ER+ or ER- tumours (Supplementary Figure 14). Next, we assessed the ability of these 3 genes in combination with the 6 genes above to identify patients with poor outcome. Irrespective of the ER status, tumours in the high-risk group had significantly lower expression levels of *JAK2, CXCR6, STAT6, IL6ST, IRF2, GBP2, TP53* and *TP63* (P ≤0.05, Figure 6A). Moreover, the results showed this 9 RNA classifier has a significant prognostic value for both ER+ (hazard ratio (HR) = 3.28 (CI: 1.76 – 6.11), p = 0.0002, Figure 6B) and ER- tumours (hazard ratio (HR) = 3.41 (CI: 1.28 – 9.06), p = 0.013, Figure 6C). We also found this 9 RNA classifer to have significant prognostic value for both ER+ (HR = 1.75 (CI: 1.44 – 2.13), p = 2.15E-08) and ER- tumours (HR = 2.04 (CI: 1.33 – 3.12), p = 0.001) in the Breast Cancer Meta-base: 10 cohorts 22K gene data on 1888 samples from the SurvExpress database [28]. These results suggest that IFN-γ signalling plays an important role in breast tumour prognosis.

## DISCUSSION

**Figure 7:**
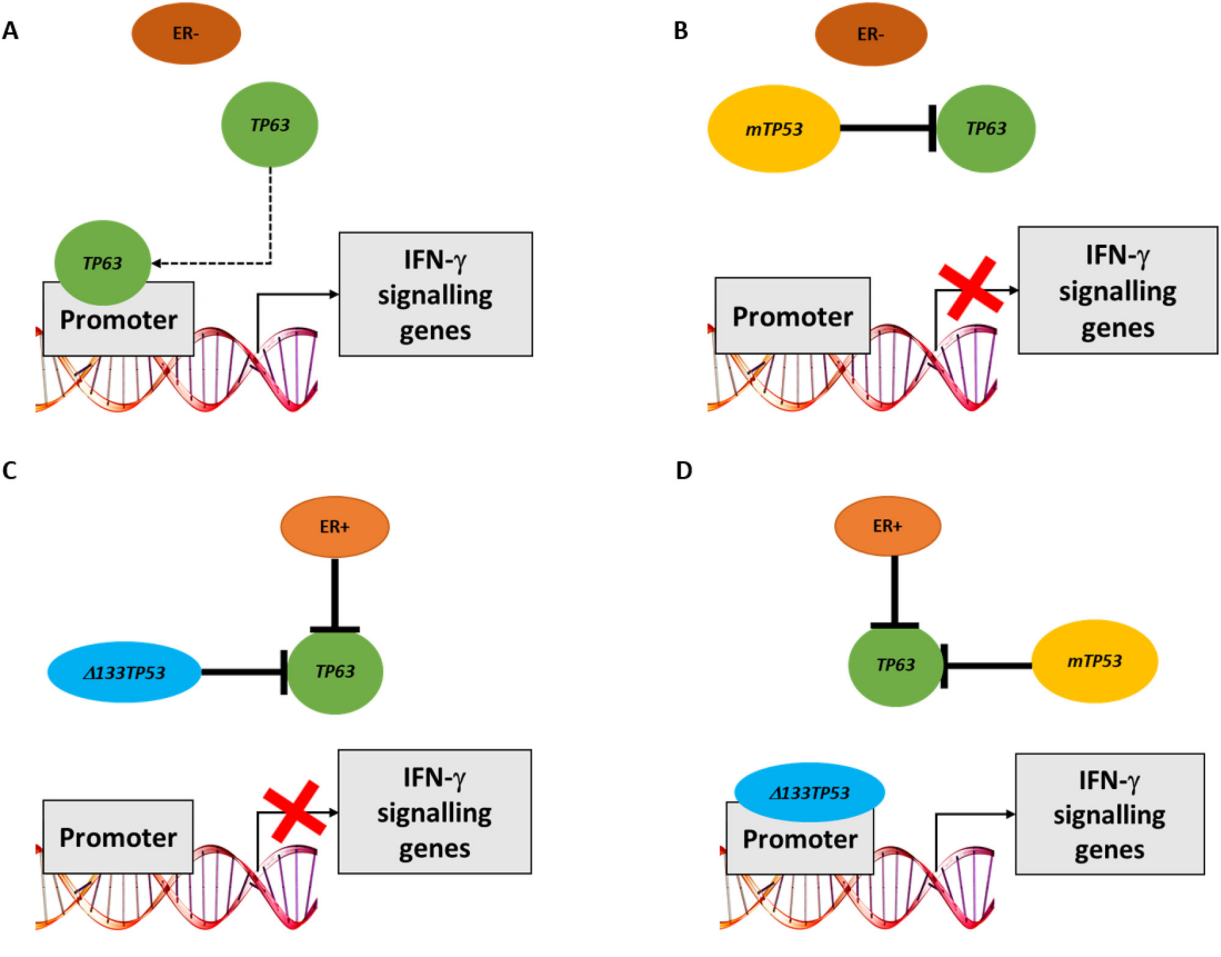
Model of how either *TP63* or *Δ133TP53* may regulate expression of IFN-γ signalling genes in different breast cancer subsets **(A)** In the absence of ER signalling and wt*TP53*, TAp63 can bind the promoter of genes involved in IFN-γ signalling. **(B)** In the absence of ER signalling and presence of m*TP53*, TAp63 is blocked by mp53 and hence cannot bind gene promoters. **(C)** In the presence of ER signalling and wt*TP53*, TAp63 transcriptional activity is blocked by binding Δ133p53. **(D)** In the presence of ER signalling and m*TP53*, mp53 can bind and inhibit TAp63. Mp53 however can no longer bind to gene promoters, thus allowing Δ133p53 to access the promoter and turn on the expression of these genes.

In this paper we investigated interactions between *TP53* mutation, expression of *TP53* isoforms and family members (*TP63* and *TP73*) in breast cancer. To do this, initially we used bioinformatic analyses that combined ER status, *TP53* mutation and RNA expression data from 3 breast cancer cohorts (TCGA, GSE3494 and GSE61725). Our results showed marked differences in signalling pathways depending on ER and *TP53* status. In ER- wt*TP53* tumours, *TP53* RNA expression was associated with cell proliferation and immune response genesets. The association was also observed with *TP53* RNA expression in the presence of frameshift and nonsense mutations (ie effectively a p53 null environment), but was lost in the presence of GOF *TP53* missense mutants. These data suggest that something other than wild type p53 is regulating these signalling pathways. One possibility we considered is the p53 family members, TAp63 and/or TAp73, as both can function as transcription factors and missense p53 mutants inactivate TAp63 and TAp73 by direct protein interaction [18, 20–22]. This is supported by our findings, where the immune response genesets are associated with *TP63* RNA expression in ER- wt*TP53* tumours, but not in the presence of m*TP53*. No association was observed with *TP73* RNA due to low expression in the tumours. Interestingly, the dominant transcriptional signature associated with *TP63* RNA expression in ER- wt*TP53* tumours was IFN-γ mediated signalling. Consistent with the bioinformatic predictions knocking down *TP63* in MDA-MB-453 (ER- w*tTP53*) cells and in MCF7 cells starved of estradiol (mimic of ER- wt*TP53*) significantly altered the expression of the candidate genes representing IFN-γ signalling but failed to alter the expression of these genes in non-starved MCF7 cells that are ER+ wt*TP53*, T47D cells that are ER+ m*TP53* or in MDA-MB-231 cells that are ER- m*TP53*. Taken together, these results suggest that TAp63 directly regulates expression of genes in the IFN-γ pathway in the ER- wt*TP53* tumour subtype (Figure 7A) but not in subtypes with m*TP53* where TAp63 is (presumably) inactivated by complexing with mp53 (Figure 7B and 7D). A limitation of this study is an inability to distinguish between the expression of *TA-TP63* or *ΔN-TP63* using current methodologies [26]. Thus, the transcriptional driver of IFN-γ signalling could be any of the *TP63* isoform family.

In contrast, in ER+ m*TP53* tumours the *Δ133TP53* isoform is positively associated with IFN-γ signalling suggesting that Δ133p53 is regulating expression of these genes. This was confirmed *in vitro* where an siRNA to *Δ133TP53* altered expression of 7/8 genes that correlated with *Δ133TP53* RNA in an ER+ m*TP53* cell line (T47D), but not ER+ wt*TP53* (MCF7) or ER- m*TP53* (MDA-MB-231) cell lines. In contrast to the bioinformatic predictions of the tumour analyses, in ER- wt*TP53* cell lines (MDA-MB-453 and MCF7 cells starved of estradiol and targeted with si-ESR1) knockdown of *Δ133TP53* resulted in reduced expression of IFN-γ signalling genes. This could be due to Δ133p53 binding to TAp63 and inhibiting it's activity as we showed in Figure 4, but as a consequence of being sequestered in this manner, there is insufficient Δ133p53 to drive transcription in ER+ wt*TP53* and ER- wt*TP53* tumours (Figure 7C). However, in the ER+ m*TP53* tumours, mp53 blocks TAp63 activity freeing up Δ133p53 to drive transcription (Figure 7D). This raises the paradox of how Δ133p53 can transactivate genes when it would also harbour mutations, and it is known that DNA binding domain mutants of Δ133p53 are defective for transactivation [29]. A possible explanation is that as the mutations in this tumour set are all heterozygous, there is one wild type *Δ133TP53* in each tumour encoding a functional, transactivation competent Δ133p53 protein.

Our results show that transcriptional upregulation of IFN-γ pathway genes is accomplished in different tumour subtypes by a distinct mechanism - either by TAp63 or Δ133p53 - with ER and *TP53* status determining which mechanism is utilized. Of significance, our data show that patients with reduced IFN-γ signalling have a shorter progression free survival. But of importance from a clinical perspective, it may be appropriate to treat these different tumour subtypes (ER- wt*TP53* and ER+ m*TP53*) with the same therapeutic strategy using for example, IFN-γ directly, or with an agonist of the pathway.

## MATERIALS AND METHODS

### Patient cohorts

Three breast cancer cohorts were used for this study. These include the Invasive Breast Cancer cohort from The Cancer Genome Atlas (TCGA), GSE3494 and GSE61725. ER status was reported based on immunohistochemistry staining and was used to stratify the tumours in ER+ and ER- groups for all three cohorts. The provisional TCGA cohort was accessed using cBioPortal for clinico-pathological data [30, 31]. Of the 1000 breast cancer patients, there was unambiguous data on the ER status determined by IHC for 421 tumours which were used for this analyses. *TP53* mutation data and Spearman’s correlation data was also accessed using cBioPortal [30, 31] for these 421 tumours. Data used for further analyses was downloaded in December 2016.

For the GSE3494 cohort, normalized gene expression data from Affymetrix U133 A and B arrays, *TP5*3 mutation status and the clinical data for 215 primary breast tumours was downloaded from the gene expression omnibus (GEO) database [32]. This microarray study was approved by the ethical committee at the Karolinska Institute, Stockholm, Sweden [33].

For the GSE61725 cohort, normalized gene expression data from Affymetrix HumanGene 1.0 ST arrays, *TP53* mutation status and the clinical data for 64 invasive ductal carcinomas was downloaded from the GEO database [32]. In this cohort there were 8 tumours that were ER+ and had mutations in *TP53*. These tumours were heterozygous for *TP53* mutations. The mutations observed in these tumours were: 742C>T - R248W, 991C>T - Q331X, 615T>A - Y205X, 602T>A - L201X, 903_904 - insC, 659A>G - Y220C, 824G>A - C275Y and 1024C>T - R342X. This study complies with the Helsinki Declaration with ethical approval from the Hunter New England Human Research Ethics Committee (approval number: 09/05/20/5.02). All patients have consented to their tissue and clinical information being used in this study [10].

### Cell culture

MCF7 and MDA-MB-231 cells (American Type Culture Collection, ATCC) were cultured in Phenol free Dulbecco's Modified Eagle Medium (DMEM, Life Technologies or Sigma) supplemented with 10% fetal bovine serum (FBS) in a 37 °C humidified incubator at 5% CO2. T47D cells (ATCC) were cultured in Phenol free RPMI-1640 media containing insulin (5 μg/ml) and 10% FBS in a 37 °C incubator at 10% CO_2_. MDA-MB-453 cells were cultured in Leibovitz L-15 media containing 10% FBS, 1% GlutaMax in a 37 °C incubator at 10% CO_2._ The SaOS2 and H1299 cells were obtained from Cell Bank Australia and were maintained in DMEM supplemented with 10% FBS in a humidified incubator at 5% CO2 and 37°C. For hormone depletion, cells were cultured for 3 days in phenol-free media supplemented with 10% charcoal dextran (Sigma)-treated FBS. Hormone-depleted cells were treated with 17-β-estradiol (Sigma) at a final concentration of 100 nM for 24 hours. All cell lines were validated for authenticity by CellBank Australia using STR profiling (http://www.cellbankaustralia.com).

### siRNA transfection

Cells were reverse transfected with stealth modified 25bp duplex siRNAs targeted to *TP63* (HSS112631), *Δ133TP53* (5′-UGUUCACUUGUGCCCUGACUUUCAA-3′) [34] and *ESR1* (HSS103377) [35] and a scrambled control siRNA (si-Ctrl 5’ - CCACACGAGUCUUACCAAGUUGCUU-3′) [36] from Invitrogen. The control siRNA has no known human mRNA targets and has been used in previous studies as a control [36]. Stealth siRNAs were transfected at a final concentration of 10 nM using Lipofectamine RNAiMax (Invitrogen). Both siRNAs and RNAiMax were diluted in medium without serum. After 10 minutes at room temperature, the diluted RNAiMax was added to the siRNAs, and the mixture was incubated for a further 15 minutes. The lipoplexes formed were added to cells. After overnight transfection, the culture medium was replaced with phenol-free media supplemented with 10% FBS until the cells were harvested at 72h. All transfections were performed in triplicate.

### Co-immunoprecipitation

Co-immunoprecipitation experiments were carried out using either SaOS2 cells stably expressing Δ133p53 (SaOS2/Δ133) [24] or the vector control (SaOs2/vector) cells. Cells were grown to 80% confluence, before being lysed in non-denaturing conditions. An agarose conjugated pAb240 antibody was added to the lysate and incubated for 1 h at 4°C, and the immunoprecipitates were collected using a microspin column. After electrophoresis on 10% SDS-PAGE and transfer, Δ133p53 was detected with the same antibody. To determine whether Δ133p53 interacted with TAp63 or TAp73, the lysates were incubated with either p63 (4A4, Abcam) or p73 (E-4, Santa Cruz) agarose conjugated antibodies, immunoprecipitated as above and immunoblotted with pAb240 (Santa Cruz).

### Colony forming assays

H1299 cells were co-transfected with expression plasmids for TAp63 or TAp73, along with increasing concentrations of a Δ133p53 plasmid, and a plasmid expressing the G418 antibiotic resistance marker (1/10th of the total amount of DNA) using FuGENE6 (Promega, USA). Twenty four hours post transfection cells were trypsinised and seeded into 12 well plates at low density and after 24h of further incubation, 1 mg/ml G418 was added to the media and the cells were cultivated for 10 days. Surviving colonies were then stained with 3% crystal violet and counted.

### RNA extraction, cDNA synthesis and reverse transcriptase – quantitative PCR (RT-qPCR)

Total RNA was isolated using either the Nucleospin RNA Isolation kit (Machery Nagel) or the RNeasy Mini Kit (Qiagen). For tumour samples, the expression of the *Δ133TP53* was determined by reverse-transcribing 660 ng of total RNA into first strand cDNA using the High Capacity cDNA Reverse Transcription Kit (Life Technologies). Real-time PCR analysis was performed in triplicate using TaqMan^®^ Universal PCR mix (Life Technologies) on 20 ng (tumour samples) of cDNA, with results quantified on a 7500 real-time PCR system (Life Technologies). Reference assays used included the Taqman Gene Expression Assays for β-actin (Human *ACTB* Endogenous Control, assay ID: Hs99999903_m1) and β2-microglobulin (assay ID: Hs99999907_m1). The primer/probes have been previously reported [10].

For cell line experiments 0.5 μg of total RNA was reverse-transcribed into first strand cDNA using the Superscript III first strand synthesis system (Life Technologies) or qScript cDNA SuperMix (Quanta Biosciences). RT-qPCR was performed as follows: 100 ng of cDNA was added to 10 μl of SYBR Premix Ex Taq II (Ti RNase H Plus) (Takara Bio) and 200 nM of each primer, in a final volume of 20 μl; the PCR product was run on the Roche LightCycler^®^ 480 (Roche); and RT-qPCR was performed for each sample with each primer pair in triplicate. Primer sequences for each primer pair has been provided in Supplementary Table 1. Reference genes used were human *ACTB* and *GAPDH*. Relative transcript abundance from RT-qPCR was calculated using the equation:

*Amplification Efficiency*^ −(geometric mean reference gene Threshold Ct−gene Threshold Ct)

### Correlation and survival analyses

Breast tumours from each cohort were divided into 4 subgroups based on ER status and *TP53* mutations: ER+ wild type (wt)*TP53*, ER+ mutant(m)*TP53*, ER- wt*TP53* and ER- m*TP53*. Spearman’s correlation analysis was then performed to identify the RNAs associated with *TP53* (201746_at – GSE3494; 8012257 – GSE61725), *TP63* (207382_at – GSE3494; 8084766 – GSE61725) and *TP73* (220804_s_at – GSE3494; 7897179 – GSE61725) RNA expression using the cor.test() function in R with the method set to “spearman” [37], unless specified otherwise. Correlation coefficient cutoff of ± 0.4 in each of the subgroups was used for gene set enrichment analyses. To overcome the difference in the number of genes that passed this cut off in the three cohorts, we took an intersection of the results between these cohorts, each assayed using different methods. Enrichment of gene sets within the input list was determined using the overrepresentation test with default settings and Bonferroni correction in the PANTHER database [16]. The number of overlapping genes between the three cohorts was determined using Venny, an online tool used to draw Venn diagrams [38].

RNA-sequencing analyses of 502 breast cancer patients from the TCGA cohort were collected from the SurvExpress web resource (http://bioinformatica.mty.itesm.mx/SurvExpress) which includes datasets from GEO [32] and TCGA [28]. Level 3, log2 transformed RNA-seq counts obtained from TCGA were used as RNA expression level of the selected 6 candidate genes representing IFN-γ signalling. 6 RNA candidates that were representative IFN-γ signalling, associated with *TP63* or *Δ133TP53* RNA expression and validated *in vitro* (Figure 5) were used to assess clinical outcome along with RNA expression of *TP53*, *TP63* and *IFN-γ*. These 6 candidates included *JAK2*, *STAT6*, *IRF2*, *CXCR6*, *IL6ST* and *GBP2*. The samples were stratified by ER status and then split into two risk groups of the same size and the prognostic index estimated using the Cox model whereby the beta coefficients were multiplied by gene expression values. Significance in the differences of the RNA expression values across the high and low risk groups was determined using the t-test (P < 0.05 was considered significant). Disease free survival was assessed for each risk group using Cox proportional hazards regression and Kaplan–Meier method. The difference between the risk groups was determined using log-rank test (P < 0.05 was considered significant).

## SUPPLEMENTARY MATERIALS FIGURES AND TABLES



## Abbreviations

ER: Oestrogen Receptor
*TP53*: Tumour protein p53
IFN-γ: Interferon-gamma
ER+: Oestrogen receptor positive
ER-: Oestrogen receptor negative
mTP53: Mutant tumour protein p53
TNBC: Triple negative breast cancer
TCGA: The Cancer Genome Atlas
GEO: Gene expression omnibus
DMEM: Dulbecco's Modified Eagle Medium
ATCC: American Type Culture Collection
RT-qPCR: Reverse transcriptase – quantitative PCR
DBD: DNA binding domain
GO: Gene ontology
wt: Wild type
FS: Frameshift
fsn: Frameshift and nonsense
GOF: Gain of function
NK cell: Natural killer cell

## References

[1] Murray-Zmijewski F, Lane DP, Bourdon JC (2006). p53/p63/p73 isoforms: an orchestra of isoforms to harmonise cell differentiation and response to stress. Cell Death Differ.

[2] Braithwaite AW, Royds JA, Jackson P (2005). The p53 story: layers of complexity. Carcinogenesis.

[3] Lane DP (1992). Cancer. p53, guardian of the genome. Nature.

[4] Candi E, Agostini M, Melino G, Bernassola F (2014). How the TP53 family proteins TP63 and TP73 contribute to tumorigenesis: regulators and effectors. Hum Mutat.

[5] Dominguez G, Silva JM, Silva J, Garcia JM, Sanchez A, Navarro A, Gallego I, Provencio M, España P, Bonilla F (2001). Wild type p73 overexpression and high-grade malignancy in breast cancer. Breast Cancer Res Treat.

[6] Bourdon JC (2007). p53 and its isoforms in cancer. Br J Cancer.

[7] Network CG, Cancer Genome Atlas Network (2012). Comprehensive molecular portraits of human breast tumours. Nature.

[8] Børresen-Dale AL (2003). TP53 and breast cancer. Hum Mutat.

[9] Olivier M, Langerød A, Carrieri P, Bergh J, Klaar S, Eyfjord J, Theillet C, Rodriguez C, Lidereau R, Bièche I, Varley J, Bignon Y, Uhrhammer N (2006). The clinical value of somatic TP53 gene mutations in 1,794 patients with breast cancer. Clin Cancer Res.

[10] Avery-Kiejda KA, Morten B, Wong-Brown MW, Mathe A, Scott RJ (2014). The relative mRNA expression of p53 isoforms in breast cancer is associated with clinical features and outcome. Carcinogenesis.

[11] Gadea G, Arsic N, Fernandes K, Diot A, Joruiz SM, Abdallah S, Meuray V, Vinot S, Anguille C, Remenyi J, Khoury MP, Quinlan PR, Purdie CA (2016). TP53 drives invasion through expression of its Δ133p53β variant. eLife.

[12] Buckley NE, Conlon SJ, Jirstrom K, Kay EW, Crawford NT, O’Grady A, Sheehan K, Mc Dade SS, Wang CW, McCance DJ, Johnston PG, Kennedy RD, Harkin DP, Mullan PB (2011). The DeltaNp63 proteins are key allies of BRCA1 in the prevention of basal-like breast cancer. Cancer Res.

[13] Su X, Chakravarti D, Cho MS, Liu L, Gi YJ, Lin YL, Leung ML, El-Naggar A, Creighton CJ, Suraokar MB, Wistuba I, Flores ER (2010). TAp63 suppresses metastasis through coordinate regulation of Dicer and miRNAs. Nature.

[14] Stantic M, Sakil HA, Zirath H, Fang T, Sanz G, Fernandez-Woodbridge A, Marin A, Susanto E, Mak TW, Arsenian Henriksson M, Wilhelm MT (2015). TAp73 suppresses tumor angiogenesis through repression of proangiogenic cytokines and HIF-1α activity. Proc Natl Acad Sci U S A.

[15] Silwal-Pandit L, Vollan HK, Chin SF, Rueda OM, McKinney S, Osako T, Quigley DA, Kristensen VN, Aparicio S, Børresen-Dale AL, Caldas C, Langerød A (2014). TP53 mutation spectrum in breast cancer is subtype specific and has distinct prognostic relevance. Clin Cancer Res.

[16] Mi H, Muruganujan A, Casagrande JT, Thomas PD (2013). Large-scale gene function analysis with the PANTHER classification system. Nat Protoc.

[17] Cowen LE, Tang Y (2017). Identification of nonsense-mediated mRNA decay pathway as a critical regulator of p53 isoform β. Sci Rep.

[18] Adorno M, Cordenonsi M, Montagner M, Dupont S, Wong C, Hann B, Solari A, Bobisse S, Rondina MB, Guzzardo V, Parenti AR, Rosato A, Bicciato S (2009). A Mutant-p53/Smad complex opposes p63 to empower TGFbeta-induced metastasis. Cell.

[19] Keyes WM, Vogel H, Koster MI, Guo X, Qi Y, Petherbridge KM, Roop DR, Bradley A, Mills AA (2006). p63 heterozygous mutant mice are not prone to spontaneous or chemically induced tumors. Proc Natl Acad Sci U S A.

[20] Melino G, De Laurenzi V, Vousden KH (2002). p73: Friend or foe in tumorigenesis. Nat Rev Cancer.

[21] Neilsen PM, Noll JE, Suetani RJ, Schulz RB, Al-Ejeh F, Evdokiou A, Lane DP, Callen DF (2011). Mutant p53 uses p63 as a molecular chaperone to alter gene expression and induce a pro-invasive secretome. Oncotarget.

[22] Li Y, Prives C (2007). Are interactions with p63 and p73 involved in mutant p53 gain of oncogenic function?. Oncogene.

[23] Campbell H, Fleming N, Roth I, Mehta S, Wiles A, Williams G, Vennin C, Arsic N, Parkin A, Pajic M, Munro F, McNoe L, Black M (2018). ∆133p53 isoform promotes tumour invasion and metastasis via interleukin-6 activation of JAK-STAT and RhoA-ROCK signalling. Nat Commun.

[24] Roth I, Campbell H, Rubio C, Vennin C, Wilson M, Wiles A, Williams G, Woolley A, Timpson P, Berridge MV, Fleming N, Baird M, Braithwaite AW (2016). The Δ133p53 isoform and its mouse analogue Δ122p53 promote invasion and metastasis involving pro-inflammatory molecules interleukin-6 and CCL2. Oncogene.

[25] Slatter TL, Hung N, Campbell H, Rubio C, Mehta R, Renshaw P, Williams G, Wilson M, Engelmann A, Jeffs A, Royds JA, Baird MA, Braithwaite AW (2011). Hyperproliferation, cancer, and inflammation in mice expressing a Δ133p53-like isoform. Blood.

[26] Mehta S, Tsai P, Lasham A, Campbell H, Reddel R, Braithwaite A, Print C (2016). A study of TP53 RNA splicing illustrates pitfalls of RNA-seq methodology. Cancer Res.

[27] Critchley-Thorne RJ, Simons DL, Yan N, Miyahira AK, Dirbas FM, Johnson DL, Swetter SM, Carlson RW, Fisher GA, Koong A, Holmes S, Lee PP (2009). Impaired interferon signaling is a common immune defect in human cancer. Proc Natl Acad Sci U S A.

[28] Aguirre-Gamboa R, Gomez-Rueda H, Martínez-Ledesma E, Martínez-Torteya A, Chacolla-Huaringa R, Rodriguez-Barrientos A, Tamez-Peña JG, Treviño V (2013). SurvExpress: an online biomarker validation tool and database for cancer gene expression data using survival analysis. PLoS One.

[29] Gong L, Pan X, Lim CB, de Polo A, Little JB, Yuan ZM (2018). A functional interplay between Δ133p53 and ΔNp63 in promoting glycolytic metabolism to fuel cancer cell proliferation. Oncogene.

[30] Cerami E, Gao J, Dogrusoz U, Gross BE, Sumer SO, Aksoy BA, Jacobsen A, Byrne CJ, Heuer ML, Larsson E, Antipin Y, Reva B, Goldberg AP (2012). The cBio cancer genomics portal: an open platform for exploring multidimensional cancer genomics data. Cancer Discov.

[31] Gao J, Aksoy BA, Dogrusoz U, Dresdner G, Gross B, Sumer SO, Sun Y, Jacobsen A, Sinha R, Larsson E, Cerami E, Sander C, Schultz N (2013). Integrative analysis of complex cancer genomics and clinical profiles using the cBioPortal. Sci Signal.

[32] Barrett T, Edgar R (2006). Gene expression omnibus: microarray data storage, submission, retrieval, and analysis. Methods Enzymol.

[33] Miller LD, Smeds J, George J, Vega VB, Vergara L, Ploner A, Pawitan Y, Hall P, Klaar S, Liu ET, Bergh J (2005). An expression signature for p53 status in human breast cancer predicts mutation status, transcriptional effects, and patient survival. Proc Natl Acad Sci U S A.

[34] Fujita K, Mondal AM, Horikawa I, Nguyen GH, Kumamoto K, Sohn JJ, Bowman ED, Mathe EA, Schetter AJ, Pine SR, Ji H, Vojtesek B, Bourdon JC (2009). p53 isoforms Δ133p53 and p53β are endogenous regulators of replicative cellular senescence. Nat Cell Biol.

[35] McEwan MV, Eccles MR, Horsfield JA (2012). Cohesin is required for activation of MYC by estradiol. PLoS One.

[36] Reid G, Wallant NC, Patel R, Antonic A, Saxon-Aliifaalogo F, Cao H, Webster G, Watson JD (2009). Potent subunit-specific effects on cell growth and drug sensitivity from optimised siRNA-mediated silencing of ribonucleotide reductase. J RNAi Gene Silencing.

[37] R Core Team (2015). R: A language and environment for statistical computing.

[38] Oliveros JC

