# Supplementary material for: Regulation of the interferon-gamma (IFN-γ) pathway by p63 and Δ133p53 isoform in different breast cancer subtypes
Source: Oncotarget. 2018 Jun 26;9(49):29146–61. doi: 10.18632/oncotarget.25635 (PMC6044385; doi:10.18632/oncotarget.25635)
Supplement: Supplementary file 1 [file oncotarget-09-29146-s001.pdf]

Regulation of the interferon-gamma (IFN-γ) pathway by p63 and Δ133p53 isoform in different breast cancer subtypes

SUPPLEMENTARY MATERIALS

A

ER Status	ER+	ER-	ER+	ER-	ER+	ER-	ER+	ER-	
TP53 Status	Wild type		Missense		Frameshift		Nonsense		P value
TCGA	296	24	28	22	9	19	6	10	< 0.00001
GSE3494	174	15	28	9	9	5	5	5	< 0.00001
GSE61725	28	11	3	9	1	2	4	3	.026721

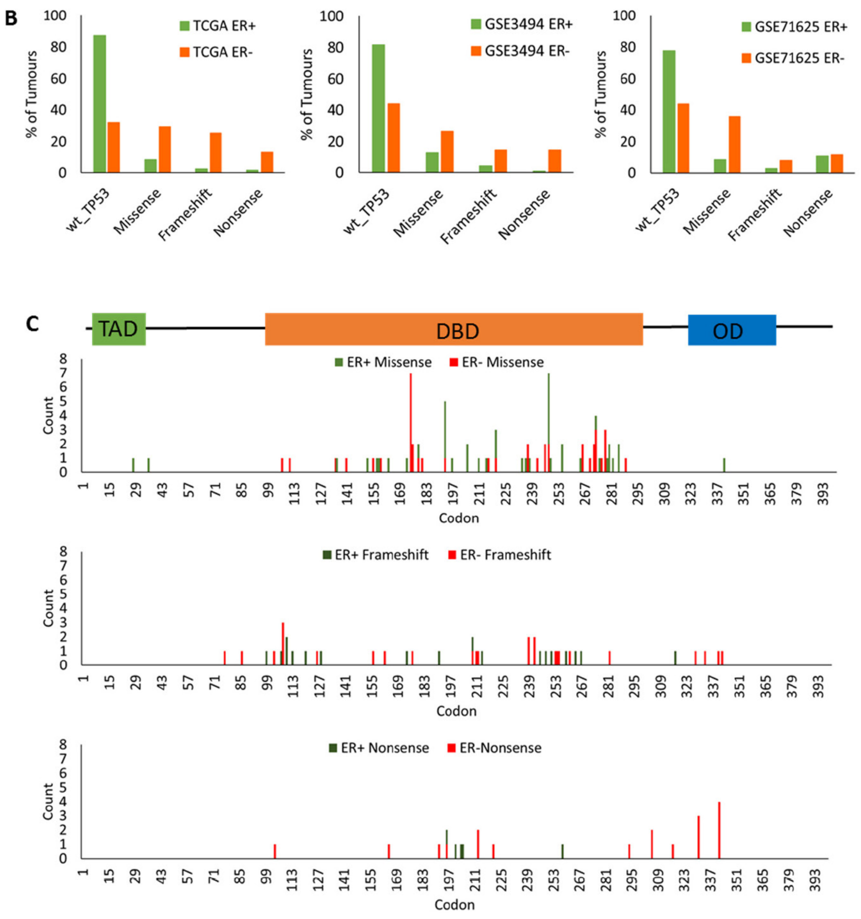

Supplementary Figure 1: Distribution of *TP53* mutation in ER-positive (ER+) and ER-negative (ER-) breast tumours. **(A)** Shows the number of tumours by ER status and *TP53* mutation in the three cohorts TCGA, GSE3494 and GSE71625. The P value is from a chi-square 2x4 contingency table of the distribution of *TP53* mutations in ER+ and ER- tumours in the three cohorts respectively. **(B)** Illustrates the distribution and percentage of ER+ and ER- tumours in the three cohorts. **(C)** Shows the combined *TP53* mutation spectrum for missense, frameshift and nonsense mutations from the three cohorts.

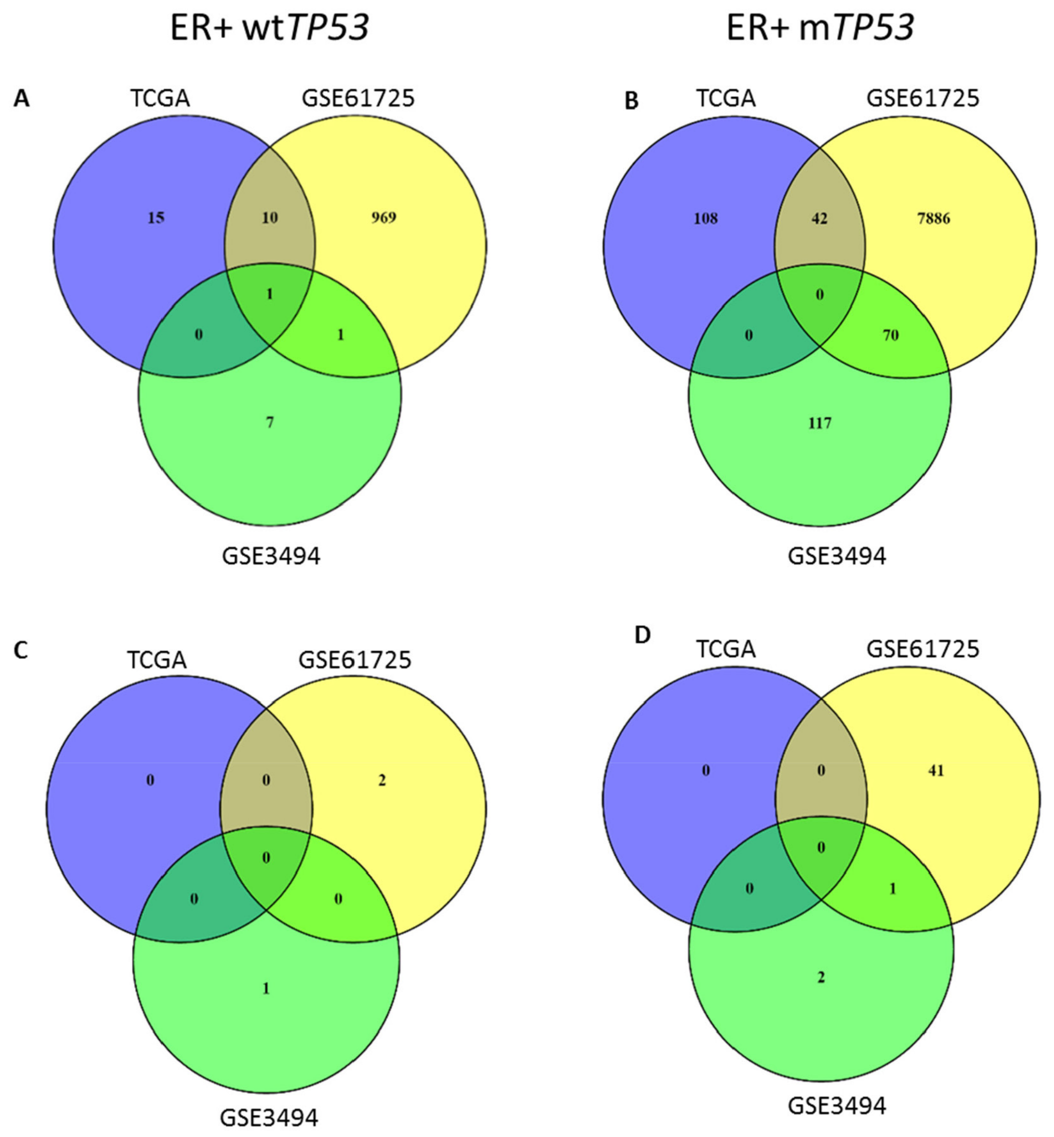

Supplementary Figure 2: RNA associated with *TP53* RNA expression do not enrich for specific biology in ER+ tumours. **(A-B)** Number of overlapping RNA associated with *TP53* RNA expression (ρ = ± 0.4) in ER+ wt*TP53*. B. Number of overlapping RNA associated with *TP53* RNA expression (ρ= ± 0.4) in ER+ m*TP53* **(C)** Number of overlapping enriched GO biological processes associated with *TP53* RNA expression (Bonferoni corrected P<0.05) in ER+ wt*TP53* **(D)** Number of overlapping enriched GO biological processes associated with *TP53* RNA expression (Bonferroni corrected P<0.05) in ER+ m*TP53*.

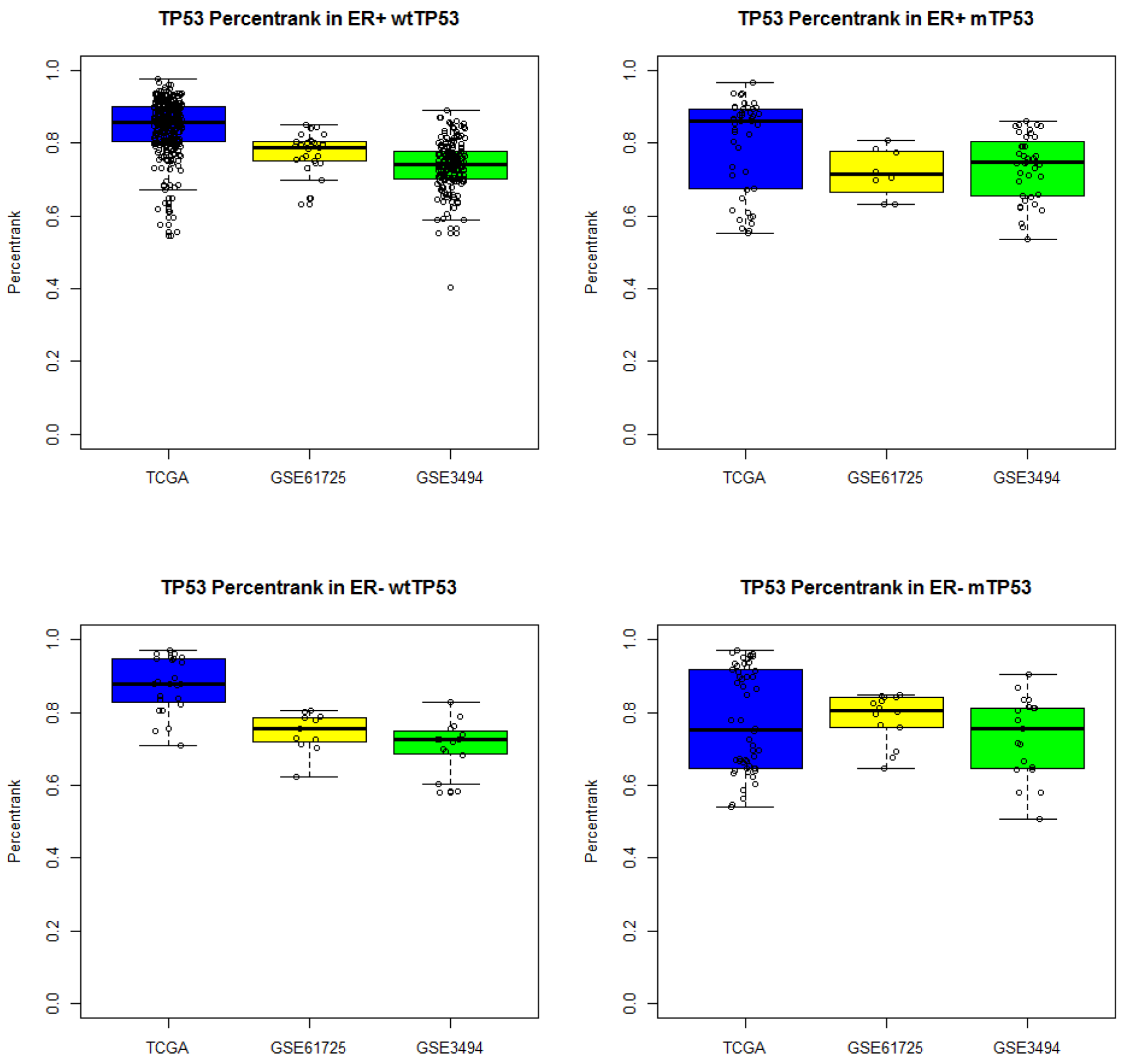

Supplementary Figure 3: Percentile rank of *TP53* gene expression in the three breast cancer cohorts. Distribution of the percentile rank of *TP53* gene expression within individual tumours in the TCGA, GSE3494 and GSE61725 cohorts. The line in the middle of each box represents the median, the top and bottom outlines of the box represent the first and third quartile respectively.

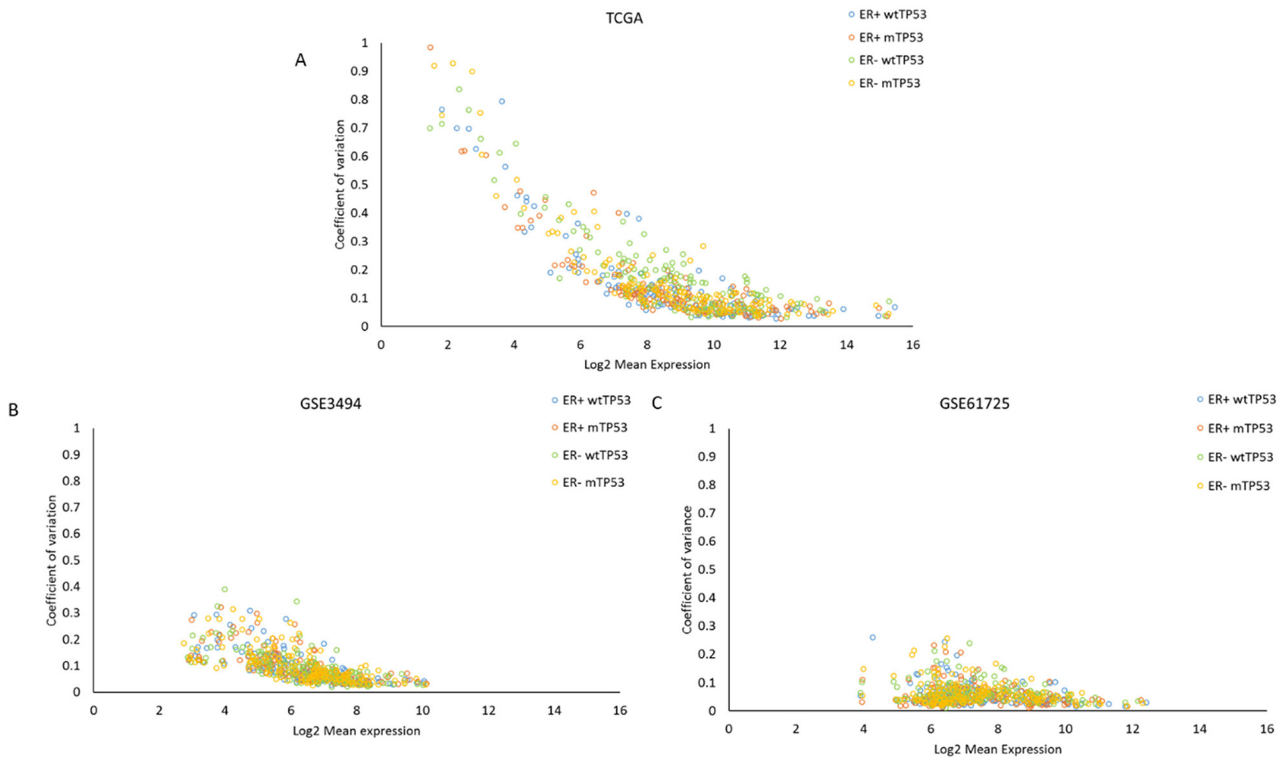

Supplementary Figure 4: Coefficient of variation of 156 RNA expression associated with *TP53* gene expression in ER- wtTP53 breast tumours between the three cohorts. The coefficient of variance versus log2 mean expression of the 156 gene associated with *TP53* expression in ER- wtTP53 breast tumours. **(A)** TCGA. **(B)** GSE3494 and **(C)** GSE61725.

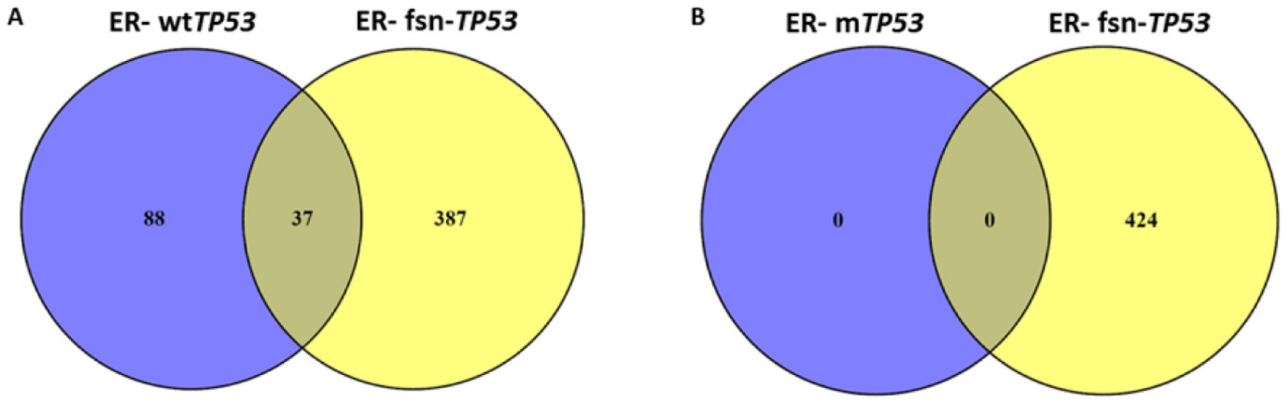

Supplementary Figure 5: Gain of function missense *TP53* mutations inhibit biological processes indirectly associated with *TP53* RNA expression. Overlap of over-represented GO biological processes (Bonferroni corrected P<0.05) associated with *TP53* RNA expression from the TCGA breast cohort in ER- tumours **(A)** A comparison of tumours with wild type *TP53* (ER- wt*TP53*, n = 24) and frameshift and nonsense mutations in *TP53* (ER- fsn*TP53*, n = 29). **(B)** A comparison of tumours with mutant *TP53* (ER- m*TP53*, n = 51) and specifically frameshift and nonsense mutations in *TP53* (ER- fsn*TP53*, n = 29).

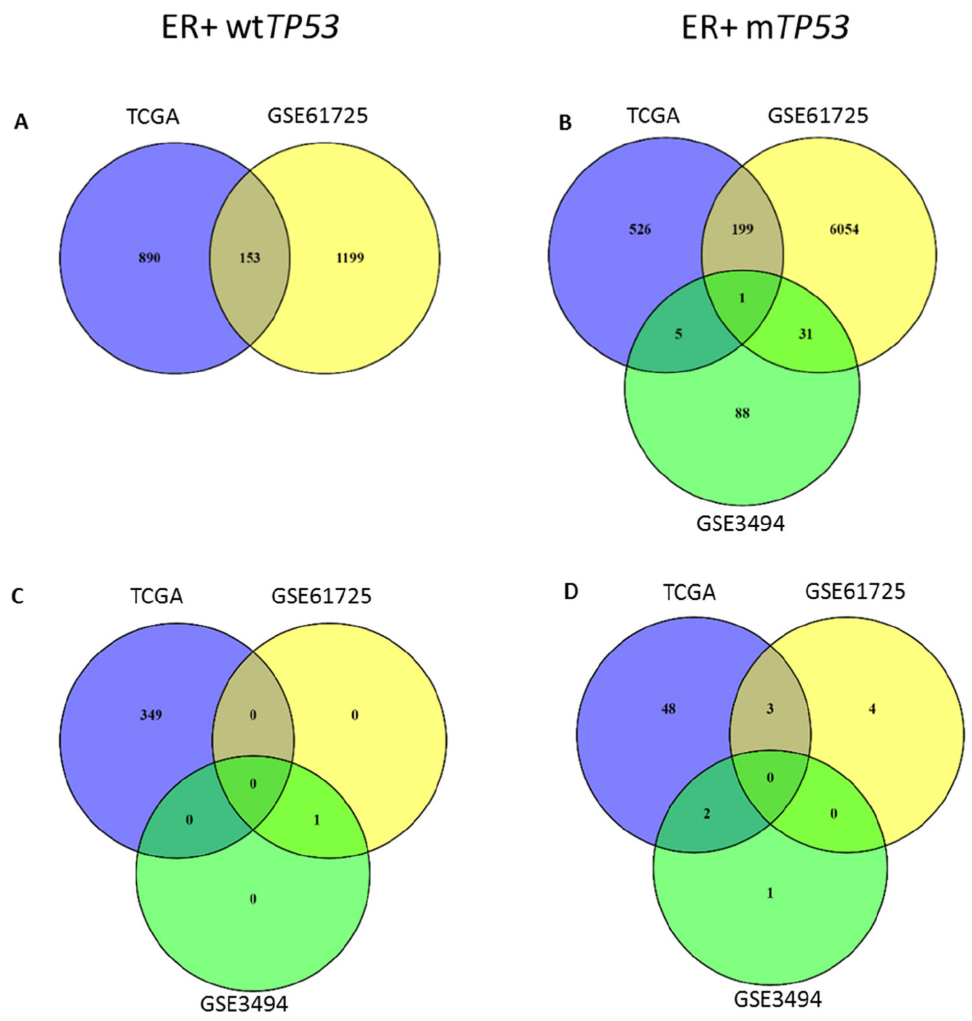

Supplementary Figure 6: RNA associated with *TP63* RNA expression do not enrich for specific biology in ER+ tumours. **(A)** Number of overlapping RNA associated with *TP63* RNA expression (ρ = ± 0.4) in ER+ wt*TP53*. **(B)** Number of overlapping RNA associated with *TP63* RNA expression (ρ= ± 0.4) in ER+ m*TP53* **(C)** Number of overlapping enriched GO biological processes associated with *TP63* RNA expression (Bonferroni corrected P<0.05) in ER+ wt*TP53* **(D)** Number of overlapping enriched GO biological processes associated with *TP63* RNA expression (Bonferroni corrected P<0.05) in ER+ m*TP53*.

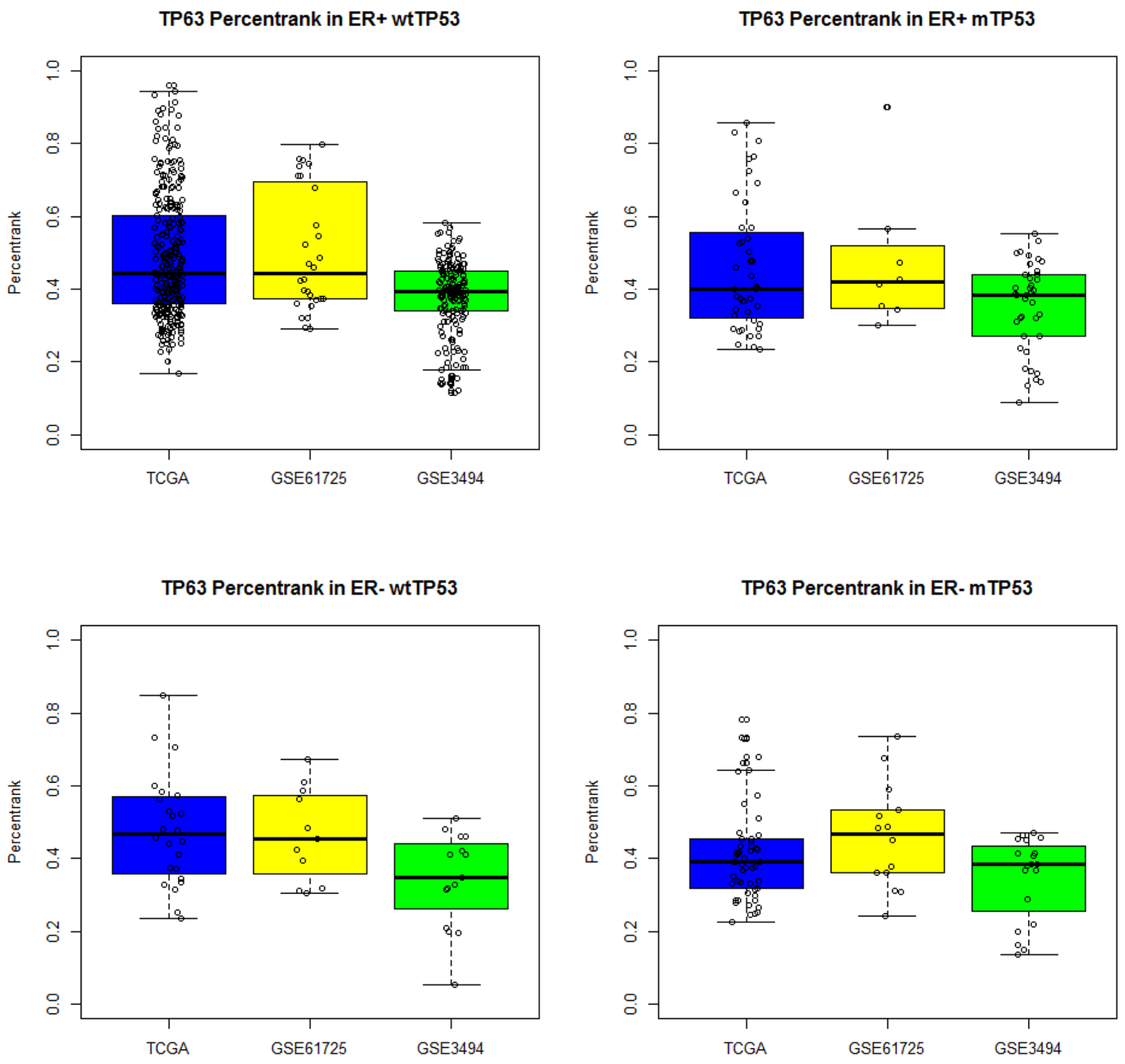

Supplementary Figure 7: Percentile rank of *TP63* gene expression in the three breast cancer cohorts. Distribution of the percentile rank of *TP53* gene expression within individual tumours in the TCGA, GSE3494 and GSE61725 cohorts. The line in the middle of each box represents the median, the top and bottom outlines of the box represent the first and third quartile respectively.

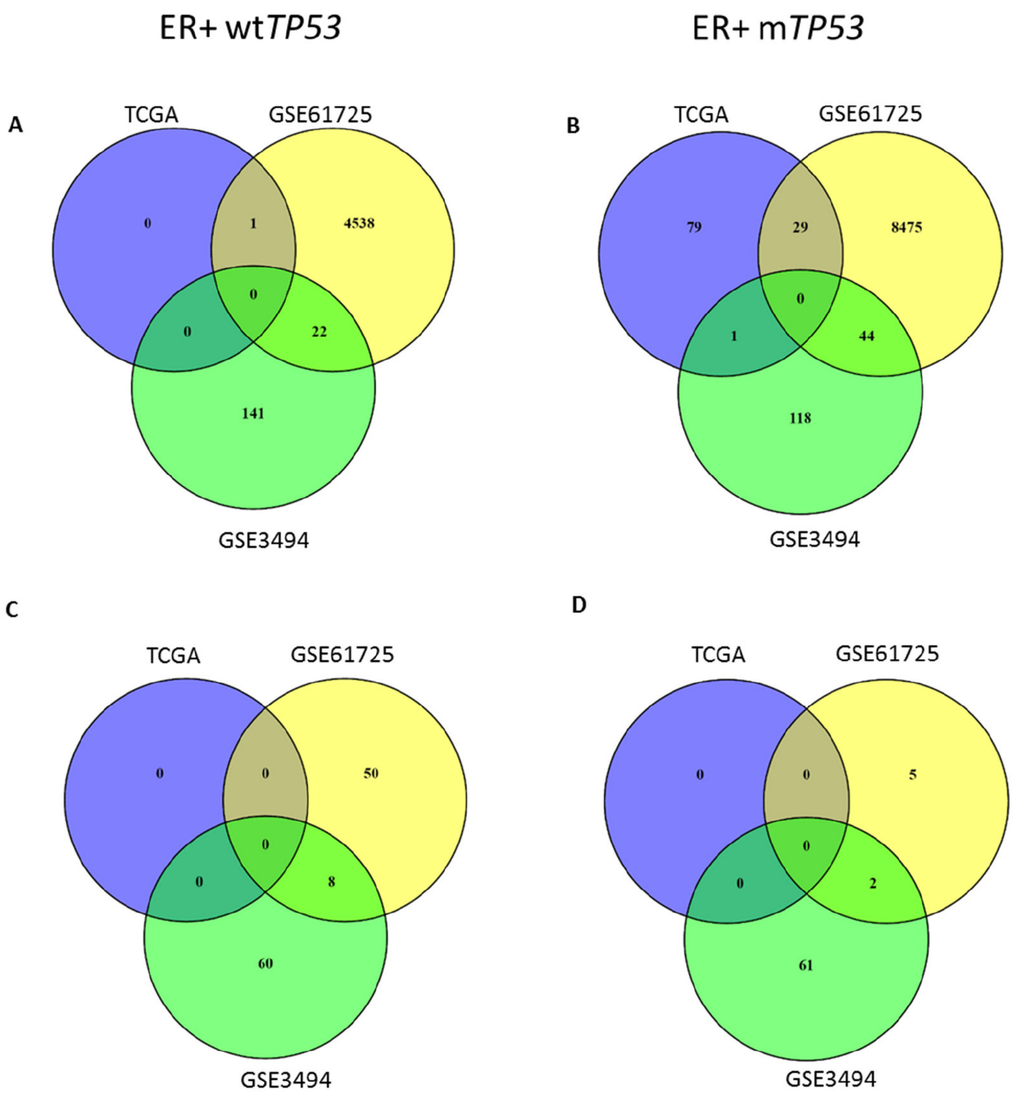

Supplementary Figure 8: RNA associated with *TP73* RNA expression do not enrich for specific biology in ER+ tumours. **(A)** Number of overlapping RNA associated with *TP73* RNA expression (ρ = ± 0.4) in ER+ wt*TP53*. **(B)** Number of overlapping RNA associated with *TP73* RNA expression (ρ= ± 0.4) in ER+ m*TP53* **(C)** Number of overlapping enriched GO biological processes associated with *TP73* RNA expression (Bonferroni corrected P<0.05) in ER+ wt*TP53*. **(D)** Number of overlapping enriched GO biological processes associated with *TP73* RNA expression (Bonferroni corrected P<0.05) in ER+ m*TP53*.

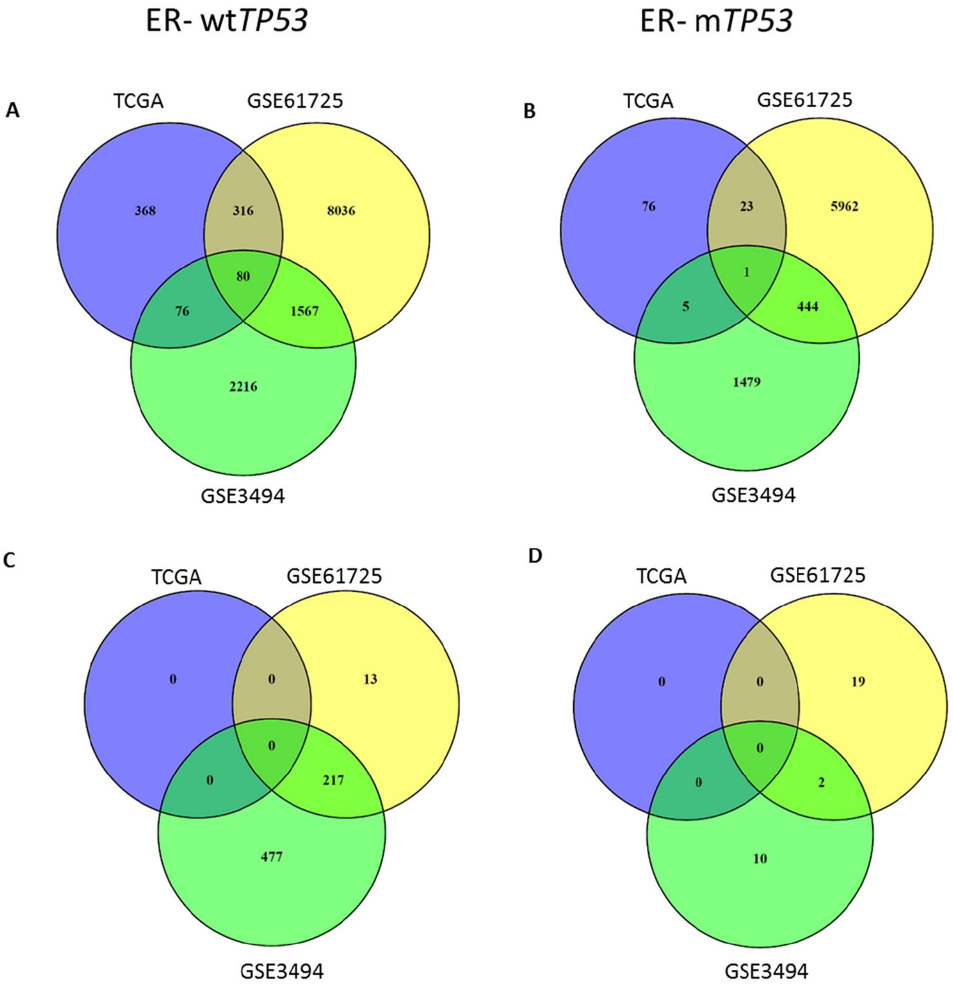

Supplementary Figure 9: RNA associated with *TP73* RNA expression do not enrich for specific biology in ER- tumours. **(A)** Number of overlapping RNA associated with *TP73* RNA expression (ρ = ± 0.4) in ER- wt*TP53*. **(B)** Number of overlapping RNA associated with *TP73* RNA expression (ρ= ± 0.4) in ER- m*TP53* **(C)** Number of overlapping enriched GO biological processes associated with *TP73* RNA expression (Bonferroni corrected P<0.05) in ER- wt*TP53* **(D)** Number of overlapping enriched GO biological processes associated with *TP73* RNA expression (Bonferroni corrected P<0.05) in ER- m*TP53*.

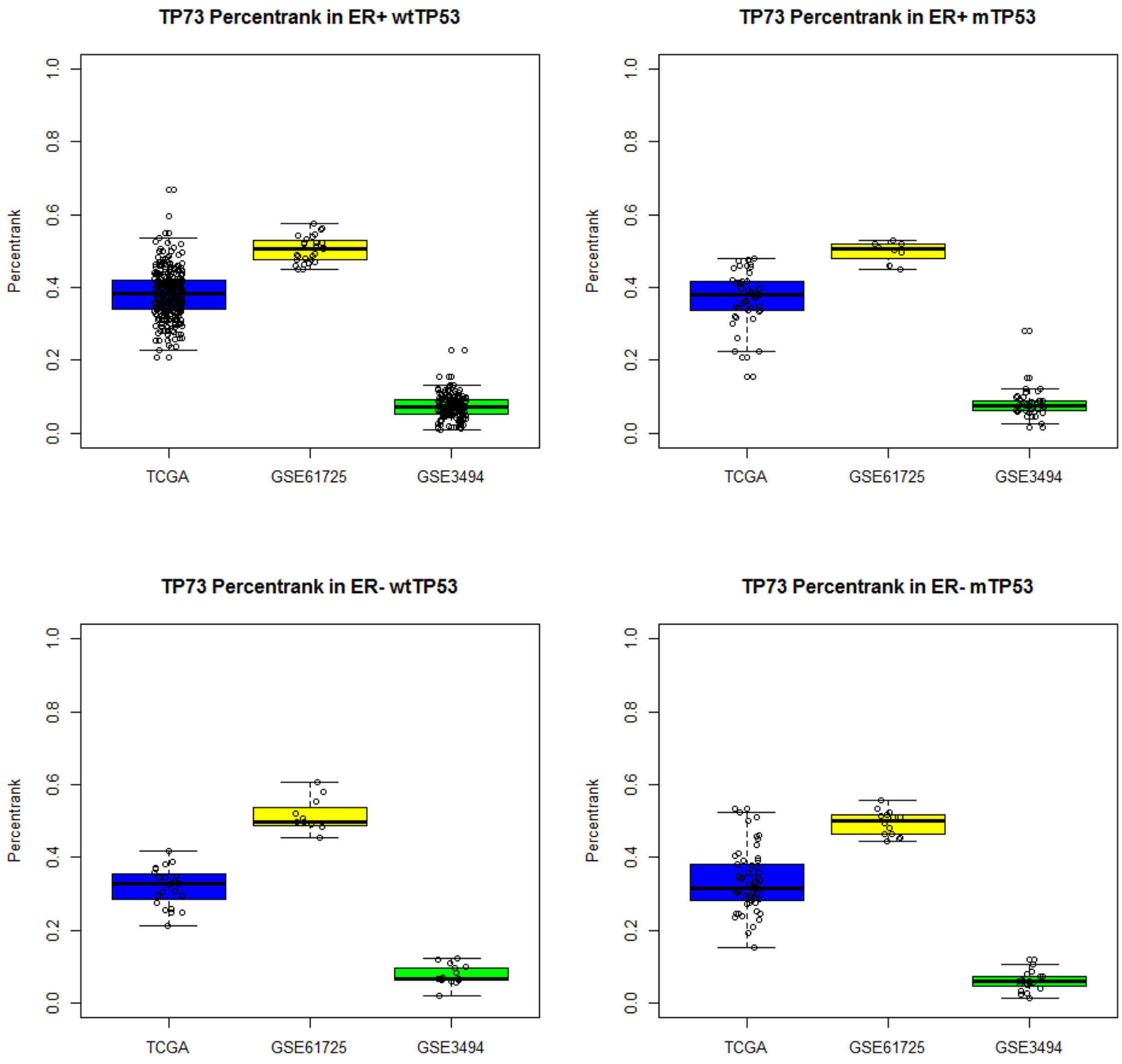

Supplementary Figure 10: Percentile rank of *TP73* gene expression in the three breast cancer cohorts. Distribution of the percentile rank of *TP53* gene expression within individual tumours in the TCGA, GSE3494 and GSE61725 cohorts. The line in the middle of each box represents the median, the top and bottom outlines of the box represent the first and third quartile respectively.

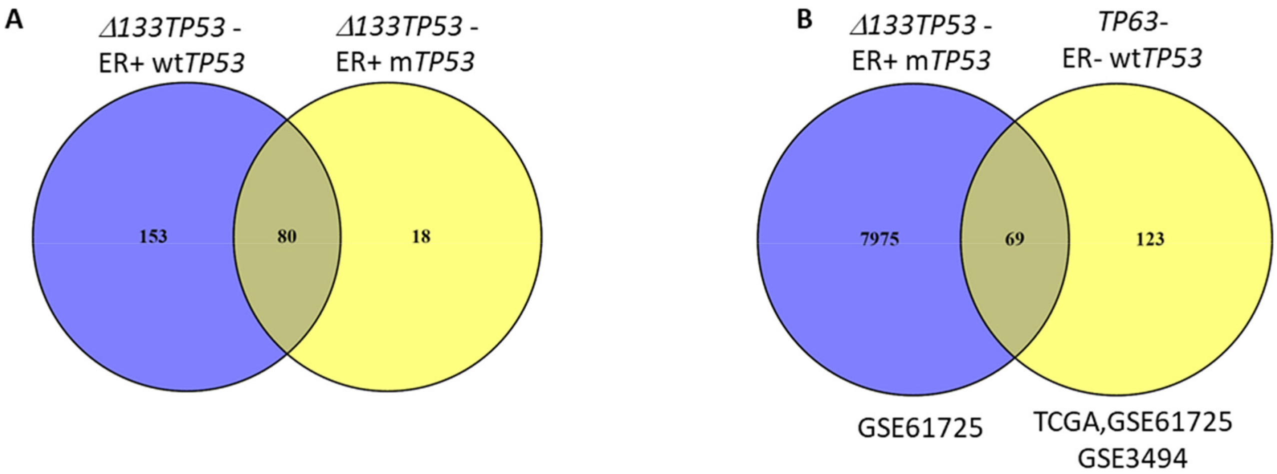

Supplementary Figure 11: *Δ133TP53* RNA expression is associated with immune response processes in ER+ m*TP53* tumours. (A) Number of overlapping and uniquely over-represented GO biological processes associated with *Δ133TP53* RNA expression (Bonferroni corrected $P<0.05$) in ER+ wt*TP53* and ER+ m*TP53* tumours from GSE61725. **(B)** Number of overlapping RNA associated with *TP63* RNA expression ($\rho = \pm 0.4$) in ER- wt*TP53* from TCGA, GSE61725 and GSE3494 and with *Δ133TP53* RNA expression in ER+ m*TP53* tumours from GSE61725.

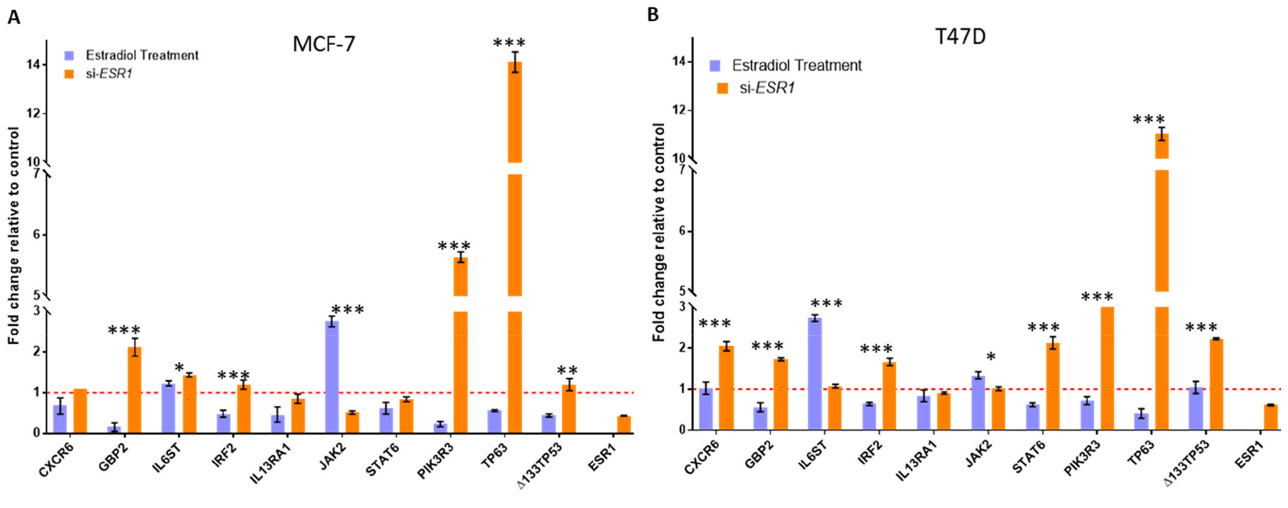

Supplementary Figure 12: Regulation of IFN-γ signalling genes by estrogen mediated signalling. (A-B) Relative fold change in the expression of candidate IFN-γ signaling pathway genes, *TP63* and *Δ133TP53*, 24h after treatment with 100nM β-oestradiol (blue) or 72h post transfection with 10nM si-ESR1 (orange) A. MCF7 cells that are ER+ wt*TP53*. B. T47D cells that are ER+ m*TP53*. A-B. Bars represent the mean fold change of expression between the treatments, with error bars representing the standard deviation. Fold change was significant if p<0.05 using a student's t-test (* < 0.05, ** < 0.005 and *** < 0.0005 respectively).

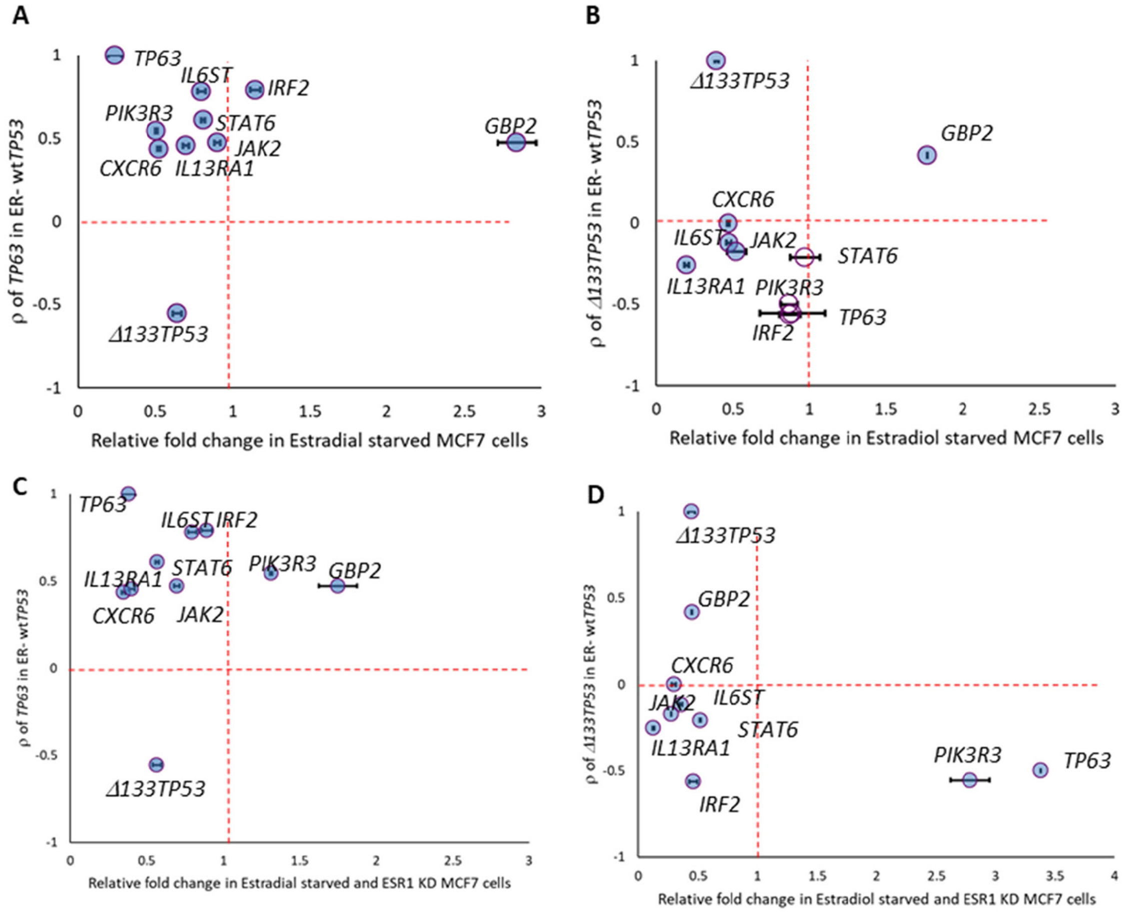

Supplementary Figure 13: Regulation of IFN-γ signalling genes by *TP63* or *Δ133TP53* in estradiol starved MCF7 cells. **(A)** Relative fold change aftersi-*TP63* treatment. **(B)** Relative fold change after si-*Δ133TP53*. **(C)** Relative fold change after si-*TP63* and si-*ESR1* treatment. **(D)** Relative fold change after si-*Δ133TP53* and si-*ESR1* treatment. A-D. Association of RNA expression of candidate IFN-γ signaling pathway genes on the y-axis and relative fold change 72h post transfection on the x-axis. Genes with positive correlation are above the horizontal dotted line and those that are negatively correlated are below the horizontal dotted line. Genes that are upregulated relative to the si-Ctrl are to the right of the vertical dotted line and downregulated genes are to the left of the vertical dotted line. Genes that are changed significantly are represented with filled colored circles, ● = significant, ○ = not significant (student's t-test, $p < 0.05$). Levels were measured in three biological replicates for each treatment.

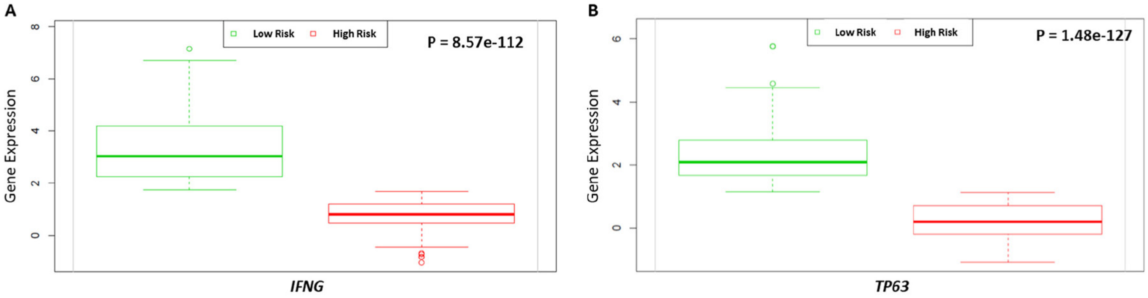

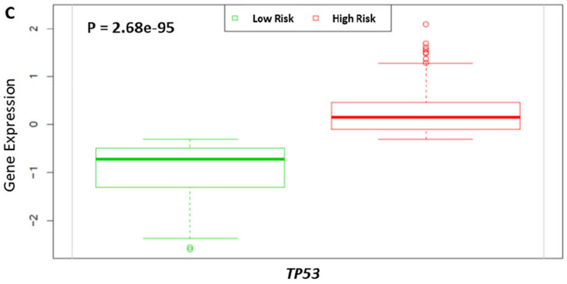

D

RNA candidates	ER+	ER-
IFNG	HR = 0.7, p = 0.26	HR = 1.53, p = 0.34
TP63	HR = 1.55, p = 0.16	HR = 0.54, p = 0.2
TP53	HR = 1.08, p = 0.82	HR = 0.73, p = 0.52
IFNG + TP63 + TP53	HR = 1.57, p = 0.14	HR = 0.72, p = 0.49

Supplementary Figure 14: RNA expression of *IFNG*, *TP63* and *TP53* does not associate with clinical outcome in breast cancer patients. Log_2 RNA expression levels of *IFNG*, *TP63*, *TP53* in high risk and low risk breast tumours from the TCGA cohort using t-test (* - p<0.05 was considered significant). **(A)** *IFNG*. **(B)** *TP63*. **(C)** *TP53*. **(D)** Summary of Cox-proportional hazard ratio of high risk versus low risk tumours stratified by expression of either *IFNG*, *TP63*, *TP53* individually or in combination from the TCGA cohort. High risk groups were those patients that had reduced disease free survival compared to low risk group patients. All analyses were done using SurvExpress.

Supplementary Table 1: Primer sequences used for ddPCR and RT-qPCR

Primer	Sequence
ACTB Forward Primer	5’- CCAACCGCGAGAAGATGA -3’
ACTB Reverse Primer	5’- CCAGAGGCGTACAGGGATAG-3’
GAPDH Forward Primer	5'-GAAGGTGAAGGTCGGAGTC-3'
GAPDH Reverse Primer	5'- GAAGATGGTGATGGGATTTC-3'
JAK2 Forward Primer	5’- GCAAGTTTTCTGTGGCCTCA-3’
JAK2 Reverse Primer	5’- ACGCATAAATTCCGCTGGTG-3’
GBP2 Forward Primer	5’- CCAAGGAAGGGGATACAGG-3’
GBP2 Reverse Primer	5’- GCTTTTTCCTTTTCTGAGAGTGAC-3’
PIK3R3 Forward Primer	5’- CCAGGGGTCATTTGAGAGGT-3’
PIK3R3 Reverse Primer	5’- CCTATGCCATTTCCACAGGC-3’
IRF2 Forward Primer	5’- GATCTCCCAGGGCTACTCAC-3’
IRF2 Reverse Primer	5’- TATCTCGTCCGTTCTGAGGC-3’
IL6ST Forward Primer	5’- GAGTCTACCCAGCCCTTGTT-3’
IL6ST Reverse Primer	5’- ACCATCACCGCCATCTACAT-3’
STAT6 Forward Primer	5’- TGCTCAGATGTGACCATGGT-3’
STAT6 Reverse Primer	5’- GGCAGCAGAGGAGGGAATAT-3’
IL13RA1 Forward Primer	5’- GCCATGAAGAGGATGCTGTG-3’
IL13RA1 Reverse Primer	5’- AAGATCACTTCCCCACTCCC-3’
CXCR6 Forward Primer	5’- CATGAATGGGTGTTTGGCCA-3’
CXCR6 Reverse Primer	5’- ATCCACAGTGATGCAGGTGA-3’
Δ133TP53 Forward Primer	5’- GCCTGAGTGACAGAGCAA-3’
Δ133TP53 Reverse Primer	5’- CGTAAGCACCTCCTGCAA-3’
TP63 Forward Primer	5’- CCTCCAACACCGACTACCCA-3’
TP63 Reverse Primer	5’- GTTTCTTCAGTTCAGTGGAATACG-3’
ESR1 Forward Primer	5’- CAGGATCTCTAGCCAGGCAC- 3’
ESR1 Reverse Primer	5’-ATGATCAACTGGGCGAAGAG-3’

Supplementary Table 2: Summary of common GO Biological processes associated with *TP53* RNA expression in ER- wt*TP53* breast tumours from TCGA, GSE61725 and GSE3494 (Statistical overrepresentation test, Bonferroni corrected P<0.05)

Sr.No.	Common GO Biological processes associated with *TP53* RNA expression in ER- wtTP53 breast tumours from TCGA, GSE61725 and GSE3494
1	leukocyte activation (GO:0045321)
2	cell activation (GO:0001775)
3	regulation of cell proliferation (GO:0042127)
4	regulation of immune system process (GO:0002682)
5	regulation of response to stimulus (GO:0048583)
6	regulation of multicellular organismal development (GO:2000026)
7	positive regulation of cellular process (GO:0048522)
8	anatomical structure morphogenesis (GO:0009653)
9	positive regulation of biological process (GO:0048518)
10	regulation of multicellular organismal process (GO:0051239)
11	regulation of developmental process (GO:0050793)
12	negative regulation of biological process (GO:0048519)
13	animal organ development (GO:0048513)
14	regulation of cell communication (GO:0010646)
15	negative regulation of cellular process (GO:0048523)
16	single-organism developmental process (GO:0044767)
17	developmental process (GO:0032502)
18	anatomical structure development (GO:0048856)
19	multicellular organism development (GO:0007275)
20	system development (GO:0048731)
21	single-multicellular organism process (GO:0044707)
22	single-organism cellular process (GO:0044763)
23	regulation of macromolecule metabolic process (GO:0060255)
24	regulation of metabolic process (GO:0019222)
25	cellular response to stimulus (GO:0051716)
26	single-organism process (GO:0044699)
27	response to stimulus (GO:0050896)
28	regulation of biological process (GO:0050789)
29	regulation of cellular process (GO:0050794)
30	biological regulation (GO:0065007)
31	cellular process (GO:0009987)
32	biological_process (GO:0008150)
33	unclassified

Supplementary Table 3: Summary of common GO Biological processes associated with *TP53* RNA expression in ER- wt*TP53* and ER- null*TP53* breast tumours from TCGA (Statistical overrepresentation test, Bonferroni corrected P<0.05)

Sr.No.	Common GO Biological processes associated with *TP53* RNA expression in ER- wt*TP53* and ER- null*TP53* from TCGA
1	regulation of hemopoiesis (GO:1903706)
2	regulation of leukocyte activation (GO:0002694)
3	positive regulation of cell proliferation (GO:0008284)
4	regulation of locomotion (GO:0040012)
5	leukocyte activation (GO:0045321)
6	cell activation (GO:0001775)
7	regulation of cell proliferation (GO:0042127)
8	regulation of immune system process (GO:0002682)
9	movement of cell or subcellular component (GO:0006928)
10	regulation of response to stimulus (GO:0048583)
11	regulation of multicellular organismal development (GO:2000026)
12	positive regulation of macromolecule metabolic process (GO:0010604)
13	regulation of localization (GO:0032879)
14	positive regulation of cellular process (GO:0048522)
15	positive regulation of cellular metabolic process (GO:0031325)
16	positive regulation of metabolic process (GO:0009893)
17	positive regulation of biological process (GO:0048518)
18	regulation of multicellular organismal process (GO:0051239)
19	regulation of developmental process (GO:0050793)
20	single-organism transport (GO:0044765)
21	regulation of signal transduction (GO:0009966)
22	regulation of biological quality (GO:0065008)
23	localization (GO:0051179)
24	negative regulation of biological process (GO:0048519)
25	single-organism localization (GO:1902578)
26	regulation of cell communication (GO:0010646)
27	negative regulation of cellular process (GO:0048523)
28	regulation of signaling (GO:0023051)
29	single-organism cellular process (GO:0044763)
30	cellular response to stimulus (GO:0051716)
31	single-organism process (GO:0044699)
32	response to stimulus (GO:0050896)
33	regulation of biological process (GO:0050789)
34	regulation of cellular process (GO:0050794)
35	biological regulation (GO:0065007)
36	cellular process (GO:0009987)
37	biological_process (GO:0008150)

Supplementary Table 4: Summary of common GO Biological processes associated with *TP63* RNA expression in ER- wt*TP53* breast tumours from TCGA, GSE3494 and GSE61725 (Statistical overrepresentation test, Bonferroni corrected P<0.05)

Sr.No.	Common GO Biological processes associated with *TP63* RNA expression in ER- wt*TP53* breast tumours from TCGA, GSE61725 and GSE3494
1	defense response (GO:0006952)
2	immune response (GO:0006955)
3	immune system process (GO:0002376)
4	single organism signaling (GO:0044700)
5	signaling (GO:0023052)
6	cell communication (GO:0007154)
7	signal transduction (GO:0007165)
8	cellular response to stimulus (GO:0051716)
9	response to stimulus (GO:0050896)
10	single-organism cellular process (GO:0044763)
11	response to chemical (GO:0042221)
12	single-organism process (GO:0044699)
13	multicellular organismal process (GO:0032501)
14	biological regulation (GO:0065007)
15	regulation of biological process (GO:0050789)
16	regulation of cellular process (GO:0050794)
17	cellular process (GO:0009987)
18	biological_process (GO:0008150)

Supplementary Table 5: Summary of common GO Biological processes represented by the 191 common RNA expression associated with *TP63* RNA expression in ER- wt*TP53* breast tumours from TCGA, GSE3494 and GSE61725 (Statistical overrepresentation test, Bonferroni corrected P<0.05)

Sr.No.	GO biological processes represented by the 191 common RNA expression associated with *TP63* RNA expression in ER- wt*TP53* breast tumours from TCGA, GSE61725 and GSE3494
1	interferon-gamma-mediated signaling pathway (GO:0060333)
2	cellular response to interferon-gamma (GO:0071346)
3	response to interferon-gamma (GO:0034341)
4	cytokine-mediated signaling pathway (GO:0019221)
5	cellular response to cytokine stimulus (GO:0071345)
6	response to cytokine (GO:0034097)
7	defense response (GO:0006952)
8	cellular response to organic substance (GO:0071310)
9	response to organic substance (GO:0010033)
10	cell surface receptor signaling pathway (GO:0007166)
11	cellular response to chemical stimulus (GO:0070887)
12	response to stress (GO:0006950)
13	signal transduction (GO:0007165)
14	cell communication (GO:0007154)
15	single organism signaling (GO:0044700)
16	signaling (GO:0023052)
17	cellular response to stimulus (GO:0051716)
18	response to stimulus (GO:0050896)
19	single-organism cellular process (GO:0044763)
20	single-organism process (GO:0044699)
21	cellular process (GO:0009987)
22	biological_process (GO:0008150)

Supplementary Table 6: Summary of common GO Biological processes associated with *Δ133TP53* RNA expression in ER+ m*TP53* breast tumours from GSE61725 (Statistical overrepresentation test, Bonferroni corrected P<0.05)

Sr.No.	GO biological processes associated with *Δ133TP53* RNA expression only in ER+ m*TP53* breast tumours from GSE61725
1	positive regulation of cell adhesion (GO:0045785)
2	lymphocyte activation (GO:0046649)
3	cell activation (GO:0001775)
4	leukocyte activation (GO:0045321)
5	regulation of response to stress (GO:0080134)
6	cell cycle process (GO:0022402)
7	cell cycle (GO:0007049)
8	positive regulation of response to stimulus (GO:0048584)
9	regulation of immune system process (GO:0002682)
10	positive regulation of signaling (GO:0023056)
11	positive regulation of cell communication (GO:0010647)
12	cell surface receptor signaling pathway (GO:0007166)
13	immune system process (GO:0002376)
14	regulation of biological quality (GO:0065008)
15	signal transduction (GO:0007165)
16	cell communication (GO:0007154)
17	signaling (GO:0023052)
18	single organism signaling (GO:0044700)

Supplementary Table 7: Summary of common GO biological processes represented by the 69 common RNA expression associated with *TP63* RNA expression in ER- wt*TP53* breast tumours from TCGA, GSE61725 and GSE3494 and *Δ133TP53* RNA expression in ER+ m*TP53* breast tumours from GSE61725 (Statistical overrepresentation test, Bonferroni corrected P<0.05)

Sr.No.	GO biological processes represented by the 69 common RNA expression associated with *TP63* RNA expression in ER- wt*TP53* breast tumours from TCGA, GSE61725 and GSE3494 and *Δ133TP53* RNA expression in ER+ m*TP53* breast tumours from GSE61725
1	interferon-gamma-mediated signaling pathway (GO:0060333)
2	cellular response to interferon-gamma (GO:0071346)
3	cytokine-mediated signaling pathway (GO:0019221)
4	positive regulation of cellular process (GO:0048522)
5	positive regulation of biological process (GO:0048518)
6	single-organism process (GO:0044699)
